# mRNA-based SARS-CoV-2 vaccines: intracellular processing and aggregation of the encoded spike protein as a mechanistic contributor to cardiac cellular stress

**DOI:** 10.3389/fimmu.2026.1635478

**Published:** 2026-02-20

**Authors:** Rolf Schreckenberg, Nadine Woitasky, Nadja Itani, Laureen Czech, Anita C. Windhorst, Malte Juchem, Christian Bär, Thomas Thum, Péter Ferdinandy, Rainer Schulz

**Affiliations:** 1 Institute of Physiology, Faculty of Medicine, Justus-Liebig University Gießen, Gießen, Germany; 2 Institute for Medical Informatics, Justus-Liebig University Gießen, Gießen, Germany; 3 Hannover Medical School, Institute for Molecular and Translational Therapeutic Strategies, Hannover, Germany; 4 Fraunhofer Institute for Toxicology and Experimental Medicine (ITEM), Hannover, Germany; 5 Fraunhofer Cluster of Excellence Immune-Mediated Diseases, Hannover, Germany; 6 Department of Pharmacology and Pharmacotherapy, Semmelweis University, Budapest, Hungary; 7 Center for Pharmacology and Drug Research and Development, Semmelweis University, Budapest, Hungary; 8 Pharmahungary Group, Szeged, Hungary

**Keywords:** cardiac inflammation, cardiotoxicity, mRNA vaccines, off-target effects, oxidative stress, SARS-CoV-2, spike (S) protein, vaccine safety

## Abstract

**Introduction:**

The trimeric spike (S) protein on the envelope of the SARS-CoV-2 virus is the primary target structure for currently approved corona vaccines. For this reason, the two mRNA-based corona vaccines Comirnaty (BNT162b2, Pfizer/BioNTech) and Spikevax (mRNA-1273, Moderna) first induce the production of a spike monomer in body cells. After enzymatic cleavage by the endoprotease furin, two S subunits are formed, which are supposed to trigger the desired immune response following secretion. Based on this concept, a preventive measure against symptomatic SARS-CoV-2 infections became available within one year of the pandemic’s onset. mRNA-based vaccines have proven highly effective in reducing severe disease and mortality. However, both the virus itself and mRNA vaccines have been associated with cardiac symptoms, which are commonly classified as myocarditis, pericarditis, or a combination thereof based on clinical presentation. Although vaccine-induced myocarditis remains a rare adverse event, recent longitudinal studies have raised questions regarding its long-term impact.

**Objective:**

To better understand the molecular mechanisms potentially involved in vaccine-associated cardiac side effects, we investigated the translation and proteolytic processing of the encoded spike monomers in human AC16 cardiomyocytes, as well as (for comparative purposes) in HEK-293 and HeLa cells.

**Results:**

In all three cell types, both BNT162b2 and mRNA-1273 produced two divergently sized monomer translation products from which one S1 subunit was formed after enzymatic cleavage. However, the number of identified S2 subunits varied between two and four depending on the cell line and mRNA used. Within a few hours, covalently bonded high-molecular complexes formed from both the spike monomers and their subunits. The arrangement of these complexes always adhered to a consistent pattern in each cell type. Particularly in AC16 cardiomyocytes, the various spike protein derivatives impaired not only cell proliferation, but also induced a pro-inflammatory response and oxidative stress. Only the secreted S1 subunit was detected as an immunogen in the supernatant of all three cell lines.

**Conclusion:**

Our findings may help to improve the safety and specificity of future mRNA platform technologies by emphasizing the importance of evaluating intracellular protein processing and the potential cellular effects of translated immunogens already during preclinical development.

## Introduction

Active immunization is one of the most important and efficient measures for preventing infectious diseases and curbing their spread. To sensitize the immune system to a specific pathogen, a precisely measured dose of a prepared immunogen is administered through conventional vaccination. Nuvaxovid, developed by Novavax, is a protein-based vaccine that uses a conventional immunization strategy to effectively combat SARS-CoV-2 infection (1–3).

At the start of the coronavirus pandemic, this previously valid definition of active immunization was expanded to include “gene-based vaccinations” for the first time. Within this new category, the mRNA-based coronavirus vaccines from Pfizer/BioNTech (BNT162b2, Comirnaty) and Moderna (mRNA-1273, Spikevax) have prevailed over the vector vaccines.

mRNA technology is based on the concept of relocating the production of immunogenic antigens into the body cells of vaccinated persons. The structure of the immunogen is determined by messenger RNA (mRNA), which is protected from degradation through encapsulation in lipid nanoparticles (LNPs). Simple endocytosis is used to incorporate the LNP-mRNA complex into the treated cells (4). However, the desired immune response only takes place after potentially cytotoxic immunogens – such as the spike (S) proteins of the SARS-CoV-2 virus – have been produced in the body cells of various organs and tissues and subsequently secreted.

Against this background, attention should be given to the currently available non-clinical biodistribution data, which indicate that LNP components and/or mRNA can distribute systemically to multiple organs. While antigen presentation via local uptake by dendritic cells and macrophages with subsequent drainage to regional lymph nodes is a key mechanism of mRNA vaccine immunogenicity (5, 6), it does not preclude systemic distribution of LNPs or vaccine mRNA. Preclinical labeled-LNP biodistribution studies and quantitative human analyses have demonstrated that LNP components and vaccine mRNA can be detected in blood and in peripheral organs (including heart, liver and spleen) (7–11); therefore both APC-mediated lymph node delivery and direct lymphatic/vascular transport of LNP/mRNA should be considered when evaluating tissue exposure and potential off-target effects. However, the extent of exposure across different cell types is heterogeneous and depends on both dose and time. While experimental data on cell type-specific consequences of endogenous spike production exist, they remain incomplete, and a comprehensive *in vivo* characterization is still lacking.

Both BNT162b2 and mRNA-1273 have been linked to adverse cardiac effects, with most cases identified clinically as myocarditis, pericarditis, or as a combination thereof (12, 13). In recent years, numerous studies have shown a statistical relationship between mRNA-based coronavirus vaccination and cardiac side effects (14–18).

In our previous study, we demonstrated hidden cardiotoxic effects from the two mRNA-based corona vaccines; these effects were attributable to spike protein expression after LNP–mRNA uptake in isolated rat cardiomyocytes and human ventricular-derived AC16 cells (19). Functionally, both mRNA-1273 and BNT162b2 caused characteristic symptoms in ventricular cardiomyocytes, in each case based on distinct pathomechanisms. mRNA-1273 induced both arrhythmic and irregular contractions due to the disruption of sarcoplasmic calcium release. BNT162b2, by contrast, led to an excessive increase in cardiomyocyte function through the chronic activation of protein kinase A (PKA). Both the impairment of the ryanodine receptor (RyR2) and the permanent activation of PKA are viewed as risk factors for sudden cardiac death, ventricular tachyarrhythmias, and contractile dysfunction (20).

In line with the findings of numerous studies, our research also indicates that the intracellular interactions of the encoded spike protein are the cause of the described symptoms and side effects. Following plasmid mediated transfection with SARS-CoV-2 spike protein encoding constructs, Clemens et al. found comparable effects on the contractile rhythm and calcium transients in human induced pluripotent stem cell-derived cardiomyocytes (hiPSC-CMs) (21).

We still lack a coherent and comprehensive model that would explain the underlying causes of the well-documented cardiac and extra-cardiac side effects that can occur as a result of mRNA-based corona vaccination. Large-scale surveillance data, such as the systematic review by Yasmin et al. covering data up to January 2022, have reported 17,636 cardiovascular events, including myocarditis, myocardial infarction, pulmonary embolism, and arrhythmias, underscoring the clinical relevance of these adverse outcomes (22). Notably, active clinical monitoring in adolescent cohorts has revealed temporary cardiovascular manifestations in nearly one in three individuals following the second dose of BNT162b2, with ECG abnormalities being one of the most common findings, while clinically evident myocarditis was a rare event. These findings suggest that mild or self-limiting cardiac involvement may be more frequent than reflected in passive reporting systems (23).

With the aim of identifying and understanding the cellular pathomechanisms involved, in this study we investigated the translation of the encoded spike protein and its intracellular processing following administration of BNT162b2 or mRNA-1273 in human AC16 cardiomyocytes and, for comparative purposes, in the human cell lines HEK-293 and HeLa.

Our findings show for the first time that the encoded spike monomers and their two subunits that arise following enzymatic cleavage by the endoprotease furin directly produce covalently bonded high-molecular complexes. We document cell-specific differences in the efficiency of spike protein formation, the intensity of the directly ensuing pro-inflammatory response, and degree of oxidative stress.

## Materials and methods

### Cultivation of human cell lines

The human cardiomyocyte cell line AC16 (#SCC109, Merck KGaA, Germany), which is derived from the fusion of primary cells from adult human ventricular heart tissues with SV40-transformed fibroblasts, was first described in 2005 (24); it was cultured according to protocol in Dulbecco′s Modified Eagle’s Medium F-12 (#D6434, Merck KGaA, Germany) together with 2 mM glutamine (#P04-80100, PAN-Biotech GmbH, Germany), supplemented with 12.5% heat-inactivated FBS (#35-079-CV, Corning Inc., USA).

The two cell lines HEK-293 (#85120602, Merck KGaA, Germany) and HeLa (#93021013, Merck KGaA, Germany) were cultured in Eagle’s Minimum Essential Medium (#M2279, Merck KGaA, Germany) together with 2 mM glutamine, 1% Non-Essential Amino Acids (#M7145, Merck KGaA, Germany), and 12.5% heat-inactivated FBS.

The three cell lines were cultured on 100 mm dishes (Falcon, Corning Inc., USA) under antibiotic-antimycotic protection (#30-004-CI, Corning Inc., USA) at 37°C in a humidified atmosphere with 5% CO_2_. For the experiments, cells between passage two and eight were plated under identical culture conditions on 60 mm dishes (Falcon, Corning Inc., USA) for SDS-PAGE/Western blot and PCR, and on 35 mm dishes (Falcon, Corning Inc., USA) for DHE and MitoSOX assays. They were consistently incubated at 60–70% confluence with identical concentrations of authentic BNT162b2 (2.0 µl or 0.2 µg RNA/ml) or mRNA-1273 (3.3 µl or 0.66 µg RNA/ml) obtained as original vials. Unloaded LNPs and LNPs containing mRNA encoding Firefly Luciferase, identical in composition and concentration to that of standard vaccine doses, were applied in the same way. To characterize the protein complexes formed post-translationally, AC16 cardiomyocytes were pre-incubated with furin inhibitor I (final concentration: 20 µM; #344930, Merck KGaA, Germany) for 1 hour prior to the application of BNT162b2 or mRNA-1273.

### Lipid nanoparticle formulation and characterization

Lipid nanoparticles were produced by microfluidic mixing using the NanoAssemblr Spark platform (Cytiva, USA). The ionizable lipids SM-102 (#Cay33474, Cayman Chemical, USA) and ALC-0315 (#890900), the phospholipid 1,2-distearoyl-sn-glycero-3-phosphocholine (DSPC, #850365), the pegylated lipids 1,2-dimyristoyl-rac-glycero-3-methoxypolyethylene glycol-2000 (DMG-PEG2000, #880151) or ALC-0159 (#880155) and cholesterol (#700100) (Avanti Polar Lipids Inc., USA) were dissolved in ethanol at a total lipid concentration of 40 mM.

For the Comirnaty formulation ALC-0315, cholesterol, DSPC and ALC-0159 were mixed in a molar ratio of 46.3/42.7/9.4/1.6%, respectively. To generate the Spikevax formulation, SM-102, cholesterol, DSPC and DMG-PEG2000 were combined in molar ratios of 50/38.5/10/1.5%, respectively. Prior to formulation, lipid mixtures were heated to 55°C for 5 minutes and then kept at room temperature. Loaded control LNPs (LNPff) were formulated with firefly luciferase modRNA dissolved in 25 mM sodium acetate buffer (pH 5.2), while empty control LNPs (LNPe) were formulated with sodium acetate buffer only. LNP formulation was performed using a flow rate ratio of 2:1 (aqueous:organic) and an N:P ratio of 6. Immediately after formulation, LNPs were diluted 30-fold in DPBS, followed by buffer exchange to 10% sucrose in DPBS via ultrafiltration using Amicon Ultra-4 centrifugal filters (Merck Millipore, USA). LNPs were sterile-filtered using 0.2 µm Acrodisc filters (#17154361, Cytiva, USA).

The encapsulation efficiency and encapsulated RNA concentration were determined using the Quant-iT RiboGreen Assay Kit (#R11490, Thermo Fisher Scientific Inc., USA). The hydrodynamic diameter and polydispersity index were determined using dynamic light scattering (Zetasizer Nano ZS, Malvern Panalytical Ltd., UK) before freezing the LNPs for long term storage at -80°C.

### Production of nucleoside-modified messenger RNA

Nucleoside-modified messenger RNA (modRNA) encoding firefly luciferase (*Photinus pyralis*) was transcribed *in vitro* from a plasmid template linearized by BtgZI (#R0703L, New England Biolabs Inc., USA). The template encoded the following components: T7 RNA promoter sequence adapted for use with CleanCap AG (#N-7113, TriLink BioTechnologies, USA) [TAATACGACTCACTATAAGG] – α-globin 5’-untranslated region – firefly luciferase open reading frame – β-globin 3’-untranslated region – segmented polyadenosine tract [60A:G:60A] (25).

The *in vitro* transcription reaction (40 mM Tris-HCl, 10 mM DTT, 2 mM spermidine, 0.002% Triton X-100, 16.5 mM magnesium acetate, 4 mM CleanCap AG, 5 mM ATP, CTP, GTP (#NU-1014L), N1-methylpseudo-UTP (#NU-890L, Jena Bioscience GmbH, Germany), 0.0002 U/µl inorganic pyrophosphatase (#EF0221, Thermo Fisher Scientific Inc., USA), 1 U/µl RNasin RNase inhibitor (N2511, Promega Corp., USA), 50 µg/ml DNA template, 8 U/µl T7 RNA polymerase (#EP0113, Thermo Fisher Scientific, USA)) was incubated for 6 hours at 37°C. The DNA template was subsequently removed by DNase digestion (15 minutes at 37°C, 100 U/ml, TURBO DNase, #AM2239, Thermo Fisher Scientific Inc., USA). The RNA was desalted with nuclease-free H_2_O using Amicon Ultra-4 centrifugal filters (#UFC801024, 10 kDa MWCO, Merck KGaA, Germany) before removing 5’-triphosphates by Antarctic phosphatase (30 minutes at 37°C, 5 U/pmol of uncapped RNA, assuming 5% uncapped transcripts, New England Biolabs Inc., USA).

To eliminate double stranded RNA byproducts, the RNA was purified using ion-pair reverse-phase high-performance liquid chromatography (IP-RP-HPLC) on a PLRP-S column (4.6 x 250 mm, 4000Å, 30 µm, column volume (CV) of 4.16 ml, #AGPL1512-5703, Agilent Technologies Inc., USA) at 62°C on an Äkta pure 25 device with a flow rate of 2.8 ml/minute (Cytiva, USA). The modRNA was loaded onto the column, washed with eluent A (100 mM TEAA in nuclease-free H_2_O) followed by a gradient of up to 20% eluent B (25% (v/v) acetonitrile; 100 mM TEAA in nuclease-free H_2_O) over 0.67 CVs. Next, the modRNA was eluted using a linear gradient from 20 to 61% eluent B over 16.2 CVs. The buffer was exchanged for 1 mM sodium citrate (pH 6.4) utilizing Amicon Ultra-4 centrifugal filters with 10 kDa MWCO.

RNA integrity and size was assessed by agarose gel electrophoresis supplemented with 0.6% (v/v) sodium hypochlorite (Carl Roth GmbH + Co. KG, Germany) (26). RNA concentration was determined by spectrophotometry (Synergy HT, Agilent Technologies Inc., USA).

### SDS-PAGE/Western blot

Cell lysis and protein extraction were performed for all cell lines according to the following protocol: Following the complete removal of the respective medium, the cells were washed twice with PBS and incubated at 4°C for 10 minutes with 200 µl of lysis buffer (#9803, Cell Signaling Technology Inc., USA). The cell lysates were then transferred to appropriate cryogenic vials, incubated on ice for an additional 20 minutes, and subjected to both mechanical homogenization (6000 rpm, 2 × 20 sec; Precellys 24, Peqlab Biotechnologie GmbH, Germany) and sonication (30 kHz, cycle: 0.7, amplitude: 60% for 30 sec; UP100H, Hielscher Ultrasonics GmbH, Germany) to ensure complete lysis. Following centrifugation for 10 minutes (14,000 × g at 4°C; Sigma 1–16K, Sigma Laborzentrifugen GmbH, Germany), the protein concentrations in the supernatants were determined photometrically using the Pierce BCA Protein Assay Kit (#23225, Thermo Fisher Scientific Inc., USA). Based on these measurements, a final loading of 5 µg of protein per well was calculated. The protein supernatants were then diluted as needed, mixed with SDS sample buffer (reducing, 2x; #S3401, Merck KGaA, Germany) according to Lämmli’s method, and heated to 85°C for 5 minutes.

For protein separation, only NuPAGE 4–12% Bis-Tris, 1.0 mm gels (#NP0321, #NP03212, #NP0323, Thermo Fisher Scientific Inc., USA) were used. Transfer to Amersham Protran 0.2 µm nitrocellulose blotting membranes (#GE10600001, Merck KGaA, Germany) was performed using XCell II Blot Modules (Thermo Fisher Scientific Inc., USA).

To detect the S1 subunit of the spike (S) monomer, the primary antibody #ABF1065 (Merck KGaA, Germany) was used in combination with the secondary antibody Goat Anti-Rabbit (#P0448, Agilent Technologies Inc., USA). The S2 subunit was detected using the two antibodies #MAB10557 (R&D Systems Inc., USA) and Goat Anti-Mouse (#P0447, Agilent Technologies Inc., USA).

Protein band visualization was performed using a Peqlab Chemiluminescence Imaging System following incubation of the membranes in SuperSignal West Pico Plus (#34580, Thermo Fisher Scientific Inc., USA). Taking into account the HiMark protein standard (#LC5699, Thermo Fisher Scientific Inc., USA), the molecular weights were determined using the software application Quantity One (version 4.6.9, Bio-Rad Laboratories Inc., USA). This software package was also used for densitometric analysis.

### Real-time PCR

After the removal of the respective medium and two washes with PBS, the cells were promptly harvested by adding 1 mL of TRItidy G (#A4051, AppliChem GmbH, Germany). After RNA isolation and photometric determination of the concentration, 1 µg of the isolated RNA was transcribed into cDNA following the manufacturer’s protocol. Following incubation with 1 U DNase/µg RNA for 15 minutes at 37°C, cDNA synthesis was carried out using SuperScript III Reverse Transcriptase (#18080093, Thermo Fisher Scientific Inc., USA).

All PCRs were performed with the CFX Connect Real-Time PCR Detection System (Bio-Rad Laboratories Inc., USA) using the iQ SYBR Green Supermix (#1708884, Bio-Rad Laboratories, Inc., USA). The cycle program consisted of an initial denaturation (95°C) of 3 minutes, followed by 45 cycles consisting of three consecutive steps: denaturation (93°C, 30 sec), hybridization (primer-specific temperature, 30 sec), and elongation (72°C, 30 sec). All reactions were performed in duplicate.

Relative gene expression differences were quantified using the ΔΔCt method described by Livak and Schmittgen. Within each cell line, the calculation consistently included the expression of the housekeeping gene HPRT (27).

To compare the relative number of mRNA copies of BNT162b2 and mRNA-1273 in the AC16, HEK-293, and HeLa cell lines, one culture dish per cell line was harvested from n=6 cell passages after 24 h of incubation. The resulting Ct values, obtained from equal amounts of transcribed RNA, were plotted separately for BNT162b2 and mRNA-1273 as relative mRNA concentrations, using HEK-293 cells as a reference (ΔCt).

For information on the primers used, see **Supplementary Table S1** in the Supplementary material.

### Fluorescence microscopic detection of superoxide using dihydroethidium and MitoSOX

Prior to application of the respective superoxide indicator, the cells were washed twice with PBS following complete removal of the cell culture medium. For the non-specific detection of superoxide using DHE, dihydroethidium (#D23107, Thermo Fisher Scientific Inc., USA) was diluted at a ratio of 1:1000 in PBS (37°C) and shielded from light. After application of the staining solution (1 ml), the cells were incubated under standard conditions for 5 minutes in an incubator. The cells were subsequently washed twice with PBS, coated with 1 mL of PBS, and analyzed with a Keyence BZ-X800 fluorescence microscope (Keyence Corporation, Japan) using a BZ-X “TRITC” filter.

To specifically detect reactive oxygen species (ROS) in mitochondria, the cells were cultured on 35 mm glass-bottom cell culture dishes (#627861, Greiner Bio-One International GmbH, Germany). 50 µg of MitoSOX (excitation at 396 nm, emission at 610 nm; #M36008, Thermo Fisher Scientific Inc., USA) was first dissolved in 12.5 µl of DMSO and then diluted at a ratio of 1:5000 in Hanks’ Balanced Salt Solution (HBSS) (#14025092, Thermo Fisher Scientific Inc., USA). After applying the indicator, the cells were incubated under standard culture conditions for 30 minutes, washed three times with HBSS, and then fixed with 4% paraformaldehyde (PFA) for 10 minutes. After removal of the PFA and a 15-minute drying phase, the cells were coated with 1 ml of HBSS; visual documentation was performed using the BZ-X “MitoSOX” filter.

At the start of each test series, all exposure parameters on the BZ-X800 microscope (e.g. exposure time, black balance, and binning) were optimized using an untreated control dish to establish baseline settings. These settings were then consistently applied when photographing all subsequent culture dishes. The image material was evaluated and analyzed using two programs: BZ-X800 Analyzer (version 1.1.30.19) and ImageJ (version 1.54d).

### Data and statistical analysis

All data are expressed as box and whisker plots. The boxes represent the lower quartile (Q1), the median, and the upper quartile (Q3); whiskers indicate the 1.5 times interquartile range (IQR). An outlier is defined as a number which is less than Q1 or greater than Q3 by more than 1.5 times the IQR. All outliers were included in the statistical analysis. Data were tested using the non-parametric Kruskal–Wallis test with subsequent pairwise Wilcoxon-Tests for independent data. A p-value less than 0.05 was considered to be statistically significant.

## Results

### Translation of the encoded spike monomer in AC16 cardiomyocytes

**Figure 1:**
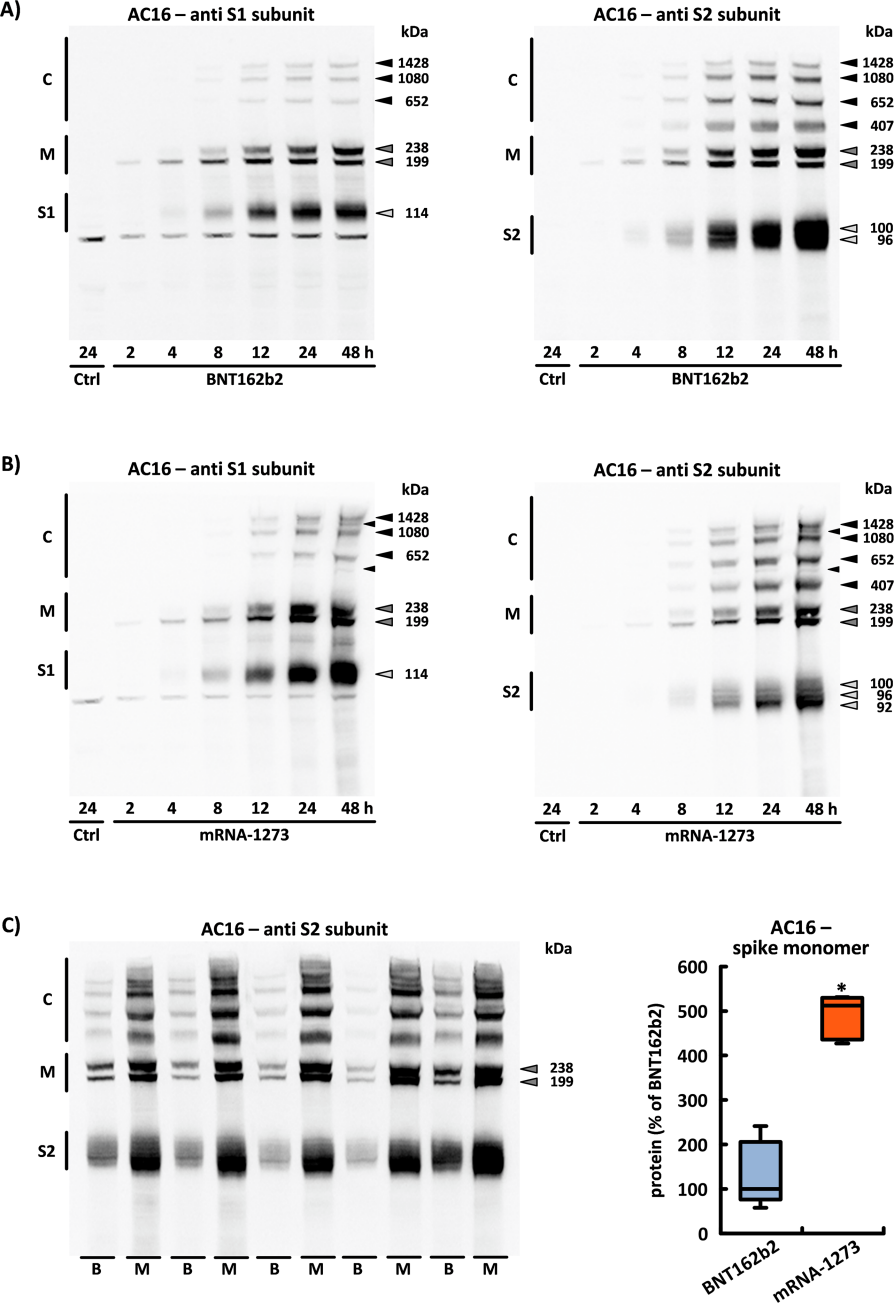
Translation of encoded spike monomers in AC16 cardiomyocytes. The figure displays all spike protein-associated products in AC16 cells detected through the S1 or S2 antibodies within 48 h following the application of **(A)** BNT162b2 and **(B)** mRNA-1273. This band pattern could be reproduced on cells from n=6 cell passages. Ctrl = untreated controls. **(C)** Original blot membrane used to quantify the amount of both translated spike monomers after 24 h incubation of BNT162b2 (B) or mRNA-1273 (M). The densitometric analysis is based on n=5 culture dishes from n=5 cell passages. *p< 0.05 *vs*. BNT162b2. (S1, S1 subunit; S2, S2 subunit; M, spike monomers; C, complexes consisting of spike monomers and its subunits).

The first spike monomers were detected in human AC16 cardiomyocytes within just two hours (h) after application of BNT162b2 or mRNA-1273, respectively. Contrary to expectations, however, two monomers with divergent molecular weights were produced by both mRNAs. The monomer with the lower molecular weight (199 kDa) could be reliably detected after 2 h, while the higher molecular weight monomer (238 kDa) could be detected after approximately 4 h (see **Figures 1A, B**).

In both cases, the translation of BNT162b2 and mRNA-1273 leads to proteins with an identical amino acid sequence and an intact furin cleavage site. Following enzymatic cleavage, the two spike subunits (S1 and S2) could be detected after approximately 4 h. With a molecular weight of 114 kDa, the S1 subunit was generated from both BNT162b2 and mRNA-1273. However, there were differences in the number of S2 subunits that occurred. In the case of BNT162b2, two S2 subunits of different sizes consistently separated from each other, while in the case of mRNA-1273, the number of S2 subunits was always three (see **Figures 1A, B**).

mRNA-1273 contains 3.3 times as much mRNA as an equivalent dose of BNT162b2 (100 µg *vs*. 30 µg). This ratio was taken into account when conducting the experiments in this study. When working with BNT162b2 (which normally has a dosage size of 300 µl), we used 2.0 µl or 0.2 µg of RNA per ml of cell culture medium. By contrast, when working with mRNA-1273 (which normally has a dosage size of 500 µl), we used 3.3 µl or 0.66 µg of RNA. We investigated the effects that these different RNA concentrations had on the synthesis rate of the spike monomers in n=5 independent experiments using one BNT162b2 and one mRNA-1273 sample, which were harvested at 24 h. Compared to incubation with BNT162b2, mRNA-1273 produced approximately 5.12x (± 0.44) more spike monomers (and their corresponding subunits) in AC16 cardiomyocytes (see **Figure 1C**).

### The intracellular formation of high-molecular weight spike protein aggregates

Approximately 8 h after application of BNT162b2 or mRNA-1273, respectively, high-molecular weight complexes arose within the cells due to the formation of covalent bonds between the spike monomer and its S1 or S2 subunits. The composition of these protein complexes was examined in n=6 independent cell passages. Both the molecular weight and band intensity of these complexes displayed a consistent pattern. Accordingly, random aggregation can be ruled out (see **Figures 1A, B**).

**Figure 2:**
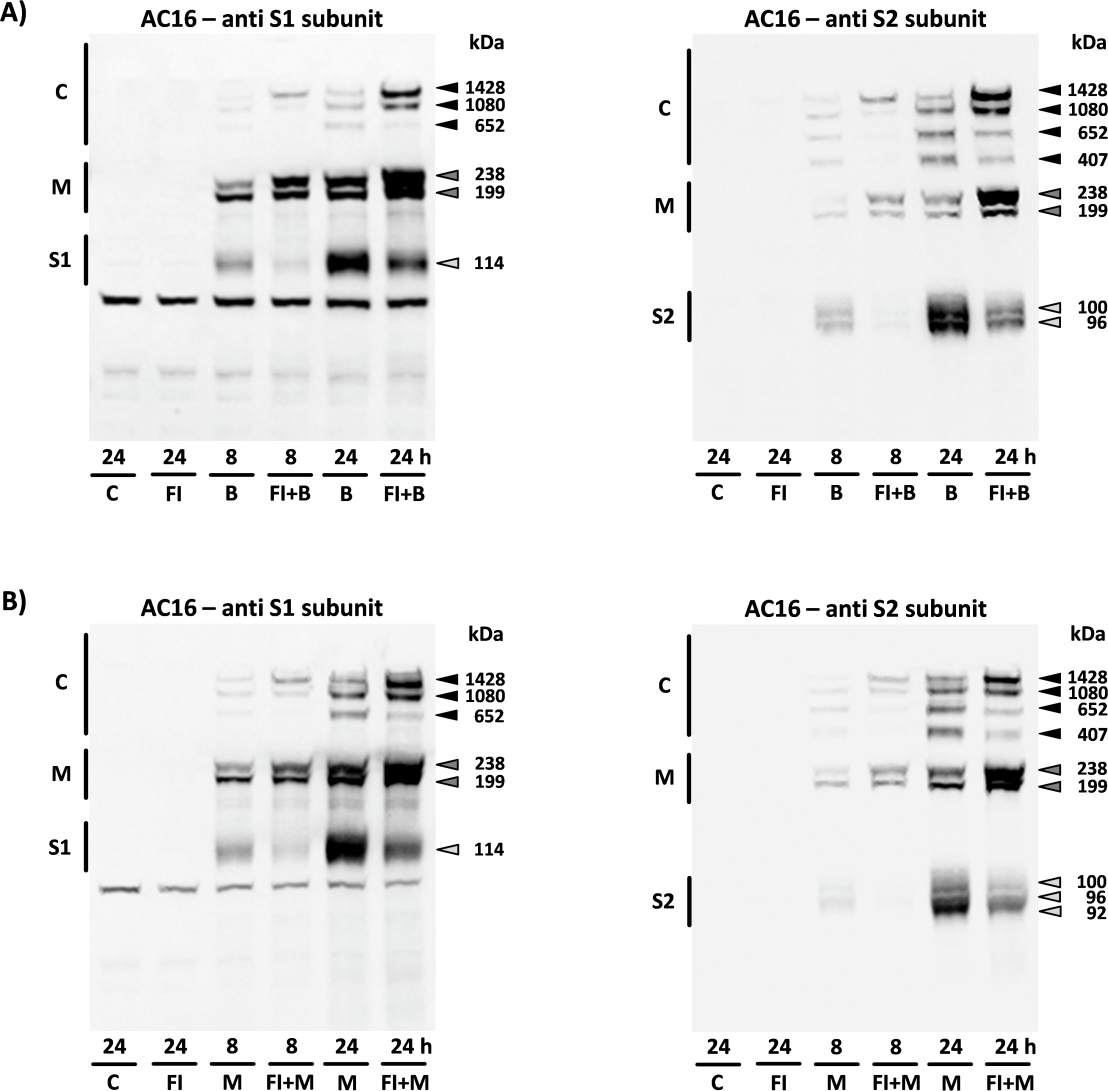
The aggregation of the spike monomers and its two S subunits. By pre-incubating the cells with furin inhibitor I (FI), it was possible to influence the band pattern exhibited by the protein complexes following translational processing. After 8 h, the “complex 407” and “complex 652” could no longer be visualized; after 24 h, they showed reduced intensity. By contrast, the FI-induced accumulation of spike monomers led to the enhanced formation of “complex 1080” and “complex 1428”. The FI effects on the band pattern were reproducibly observed in cells from n=4 cell passages following incubation with **(A)** BNT162b2 (B) and **(B)** mRNA-1273 (M). C, untreated controls, FI, cells treated with FI only. (S1, S1 subunit; S2, S2 subunit; M, spike monomers; C, complexes consisting of spike monomers and its subunits).

Due to their excellent specificity towards the S1 or S2 subunit, it was possible to conclude solely based on the antibody used that “complex 407” was composed exclusively of S2 subunits. After pre-incubation of the cells with furin inhibitor I, the formation of the S subunits was almost completely prevented over a period of 8 h, thus specifically influencing the band pattern. “Complex 407”, which was detected exclusively in connection with the S2 antibody, could no longer be observed under these conditions. “Complex 652”, previously recognized by both antibodies, was also no longer detected, and was thus composed of the S1 and S2 subunits. The two complexes with the highest molecular weight (“complex 1080” and “complex 1428”), which were also detected by both antibodies, increased significantly in intensity. Accordingly, they were formed by the accumulating spike monomers (see **Figures 2A, B**). Two further bands with a molecular weight of 538 kDa and 1286 kDa, respectively, could only be detected given optimal separation and efficient transfer, due to their low intensity (see **Figure 1B**).

**Figure 3:**
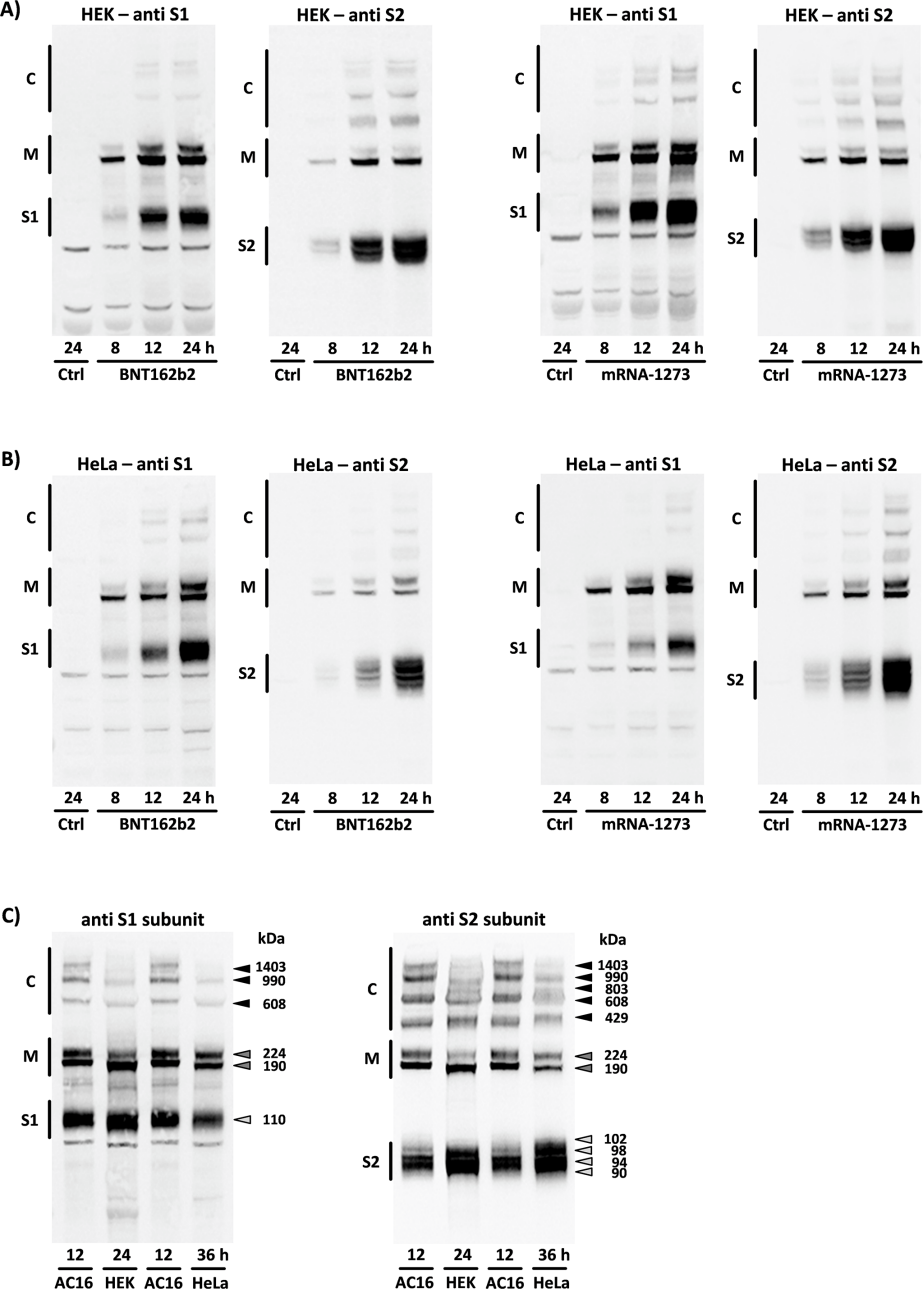
Translation of the encoded spike monomers in HEK-293 and HeLa cells. The figure displays all spike protein-associated products detected through the S1 or S2 antibodies following 8 h, 12 h, and 24 h of incubation with BNT162b2 or mRNA-1273 in **(A)** HEK-293 and **(B)** HeLa cells. The band pattern could be reproduced on cells from n=5 cell passages. Ctrl = untreated controls. **(C)** Original blot membranes showing the cell type-specific differences in the translation of the spike monomers and production of associated derivatives after application of mRNA-1273. Different incubation times were required for the purposes of comparison, due to individual translation efficiency (see **Figure 4**). (S1, S1 subunit; S2, S2 subunit; M, spike monomers; C, complexes consisting of spike monomers and its subunits).

Identical experiments with the cell lines HEK-293 and HeLa were performed to determine the extent to which the described band pattern may be specific to AC16 cardiomyocytes. After application of BNT162b2 and mRNA-1273, two spike monomers of different sizes were also formed, but in contrast to the monomers of the AC16 cells, they had a lower molecular weight of 190 and 224 kDa, respectively. The band pattern displayed by the monomers and its two subunits differed qualitatively and quantitatively from that of the AC16 myocytes. With the exception of “complex 407”, which had a higher molecular weight in the HEK and HeLa cells, all other complexes had a lower molecular weight. Furthermore, in both cell lines we observed an additional band with a molecular weight of 803 kDa that could only be detected by the S2 antibody. Regardless of the mRNA used in each case, given optimal separation, one S1 and two S2 subunits could be reliably identified in HEK-293 cells, and one S1 and four S2 subunits in HeLa cells (see **Figures 3A–C**).

### Cell type-specific differences in spike production

**Figure 4:**
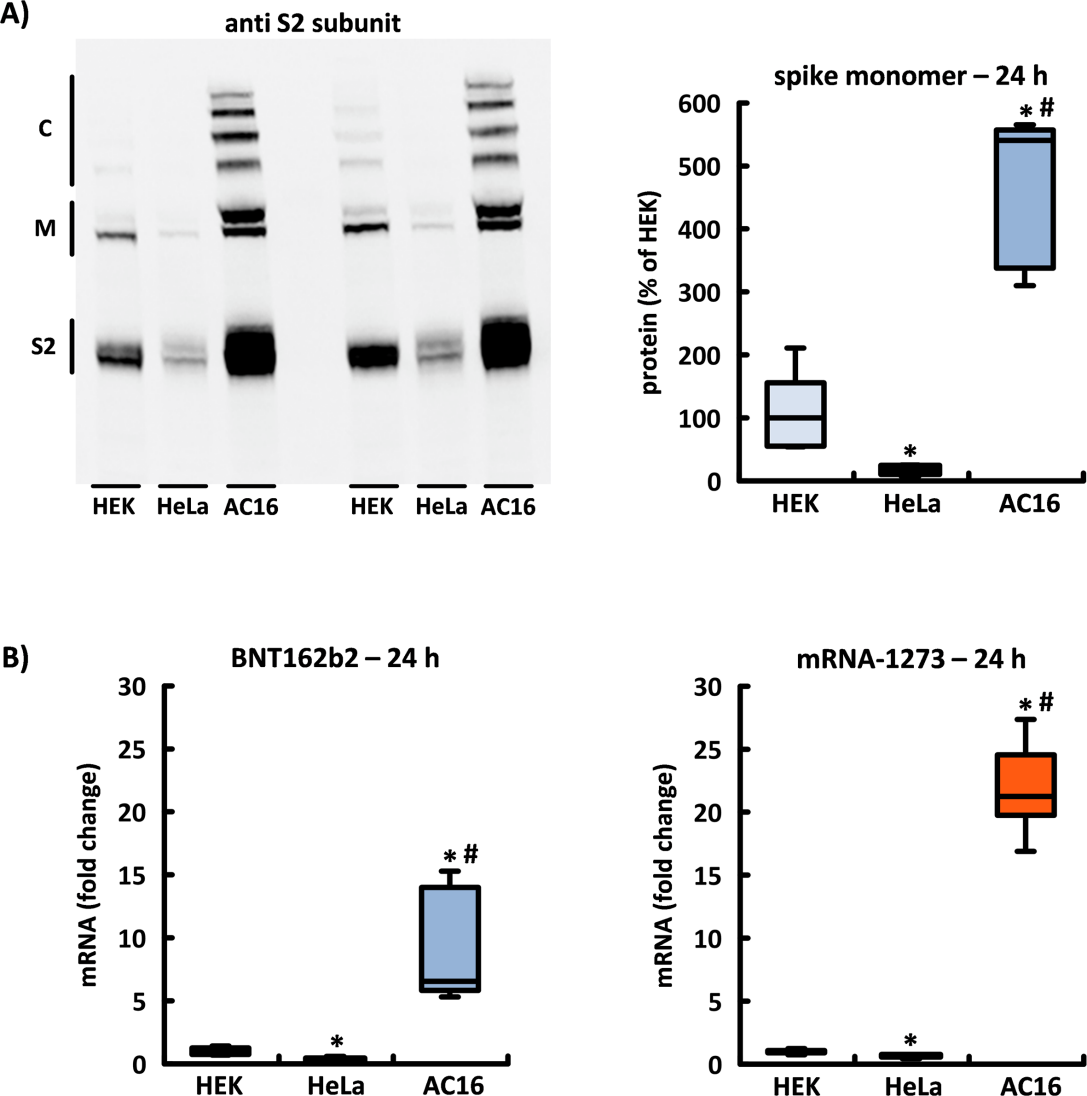
The translation efficiency of spike monomers as a function of cell type. The three cell lines exhibited divergent levels of efficiency in their production of the encoded spike monomers and subsequently of the post-translationally formed subunits and high-molecular protein aggregates. **(A)** Original blot membrane with n=2 independent experiments showing the quantitative differences in the band patterns displayed by the HEK-293, HeLa, and AC16 cells after 24 h incubation with BNT162b2. The densitometric evaluation of the two spike monomers is based in each case on n=5 culture dishes from n=5 cell passages. **(B)** The relative number of incorporated mRNA copies after 24 h incubation with BNT162b2 or mRNA-1273 in the three cell lines HEK-293, HeLa, and AC16 is shown using HEK-293 cells as a reference. The results are based in each case on n=6 culture dishes from n=6 cell passages. *p < 0.05 *vs*. HEK, #p < 0.05 *vs*. HeLa. (S2, S2 subunit; M, spike monomers; C, complexes of spike monomers and its subunits).

After application of BNT162b2 and mRNA-1273, the amount of spike protein produced indicated dramatic differences in the translation efficiency achieved in each studied cell line. In AC16 cardiomyocytes, the concentration of spike monomers and their subunits as well as of high-molecular weight complexes exceeded the corresponding concentrations in HEK-293 and HeLa cells at all time points. Of the two cell lines studied for comparative purposes, the HEK-293 cells had a band pattern of higher intensity (see **Figure 4A**).

The foregoing findings can be explained by the significant difference in the intracellular mRNA concentrations between the three cell lines. After 24 h of incubation, the number of mRNA copies of BNT162b2 in the AC16 cells was 6.6x that of HEK-293, while in HeLa cells it was 3.2x lower. Following application of mRNA-1273, there were 21.3x more mRNA copies in AC16 cells than in HEK-293, while in HeLa cells the number of copies was reduced 1.4x (see **Figure 4B**).

### The pro-inflammatory properties of the spike protein

The majority of cardiac side effects attributed to mRNA-based corona vaccination are classified clinically as myocarditis and/or pericarditis. Consistent with this side effect profile, a massive increase in IL-6 expression was detected in the AC16 cells 24 h after application of BNT162b2 and mRNA-1273. This increase was largely caused by the production of the spike protein and its high-molecular weight aggregates. mRNA-1273 increased IL-6 expression 15.08x (± 6.28), while BNT162b2 increased IL-6 expression 1.72x (± 0.15).

**Figure 5:**
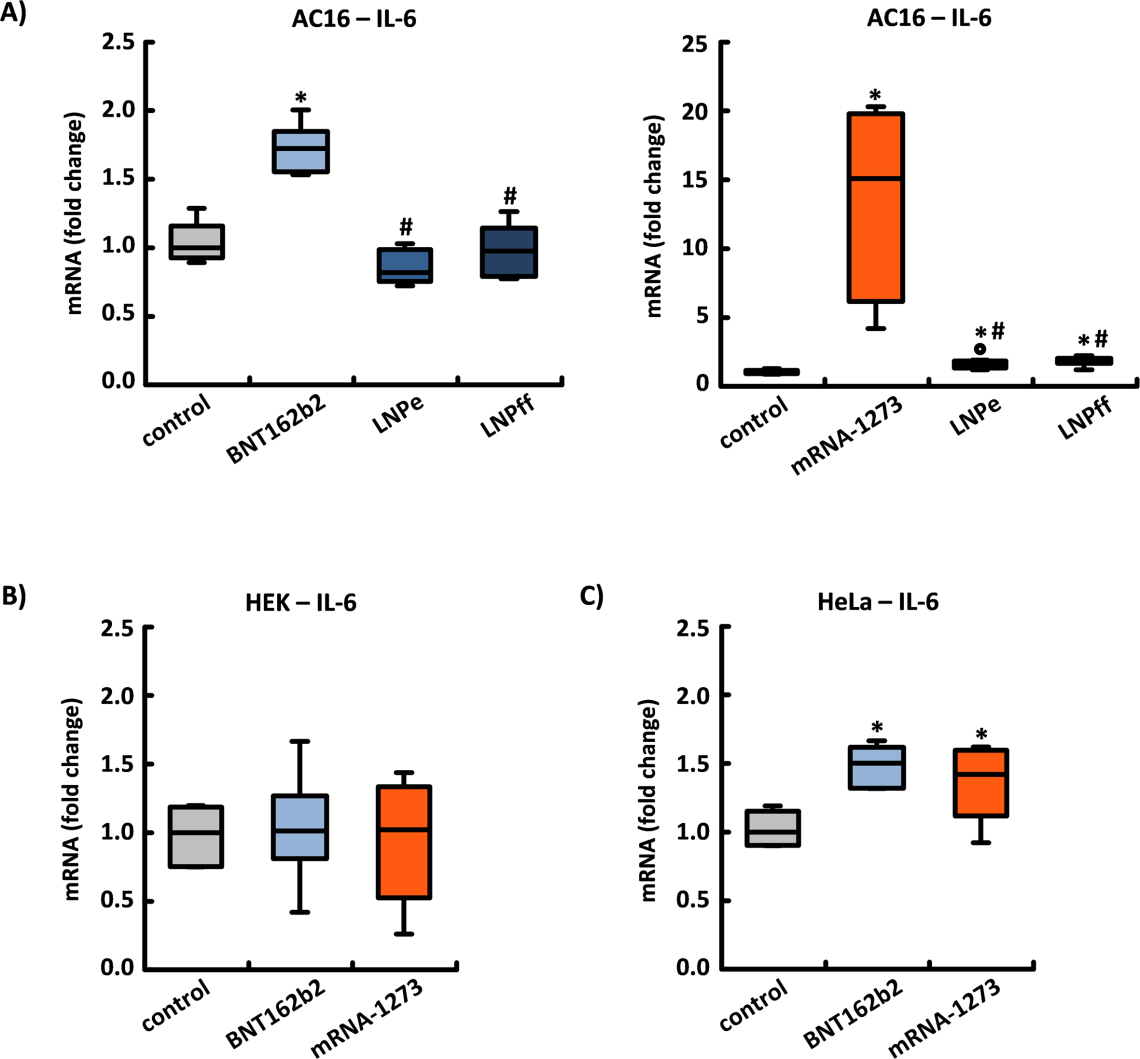
The induction of the pro-inflammatory cytokine interleukin-6 (IL-6). **(A)** Both BNT162b2 and mRNA-1273 caused a significant induction in IL-6 mRNA expression within 24 h in human AC16 cardiomyocytes. In contrast to the LNPs in BNT162b2, both unloaded LNPs (LNPe) and LNPs loaded with mRNA for Firefly Luciferase (LNPff) used in mRNA-1273 increased the expression of IL-6 by a factor of 1.51 ± 0.45 and 1.74 ± 0.29, respectively. These results are based in each case on n=8 culture dishes from n=4 cell passages. In **(B)** HEK-293 cells, neither BNT162b2 nor mRNA-1273 had any effect on IL-6 expression, whereas in **(C)** HeLa cells the application of both mRNAs led to a significant increase. These results are based in each case on n=6 culture dishes from n=3 cell passages. *p < 0.05 *vs*. control, #p < 0.05 *vs*. BNT162b2 or mRNA-1273.

Expression of IL-6 was not increased by unloaded LNPs or by LNPs loaded with mRNA for firefly luciferase, whose composition corresponded to that of BNT162b2. By contrast, the composition of the LNPs selected by Moderna induced the expression of IL-6 to a comparable extent both in the unloaded state (1.51x ± 0.45) and when loaded with firefly mRNA (1.74x ± 0.29) (see **Figure 5A**).

In HEK-293 cells, neither BNT162b2 nor mRNA-1273 had any effect on the expression of IL-6; in HeLa cells, however, both BNT162b2 and mRNA-1273 induced increased expression by a factor of 1.50 (± 0.14) and 1.42 (± 0.26), respectively (see **Figures 5B, C**).

### The influence of BNT162b2 and mRNA-1273 on the expression of RNA-binding antiviral proteins

The four identified IFIT genes code for members of a protein family that are induced by type I interferon (IFN) and viral infections as well as by the recognition of pathogen-associated molecular patterns (PAMPs). In addition, single- and double-stranded viral nucleic acids can be recognized and bound by IFIT proteins. The primary goal of IFITs is to block the translation of viral RNA and initiate its degradation (28, 29).

**Figure 6:**
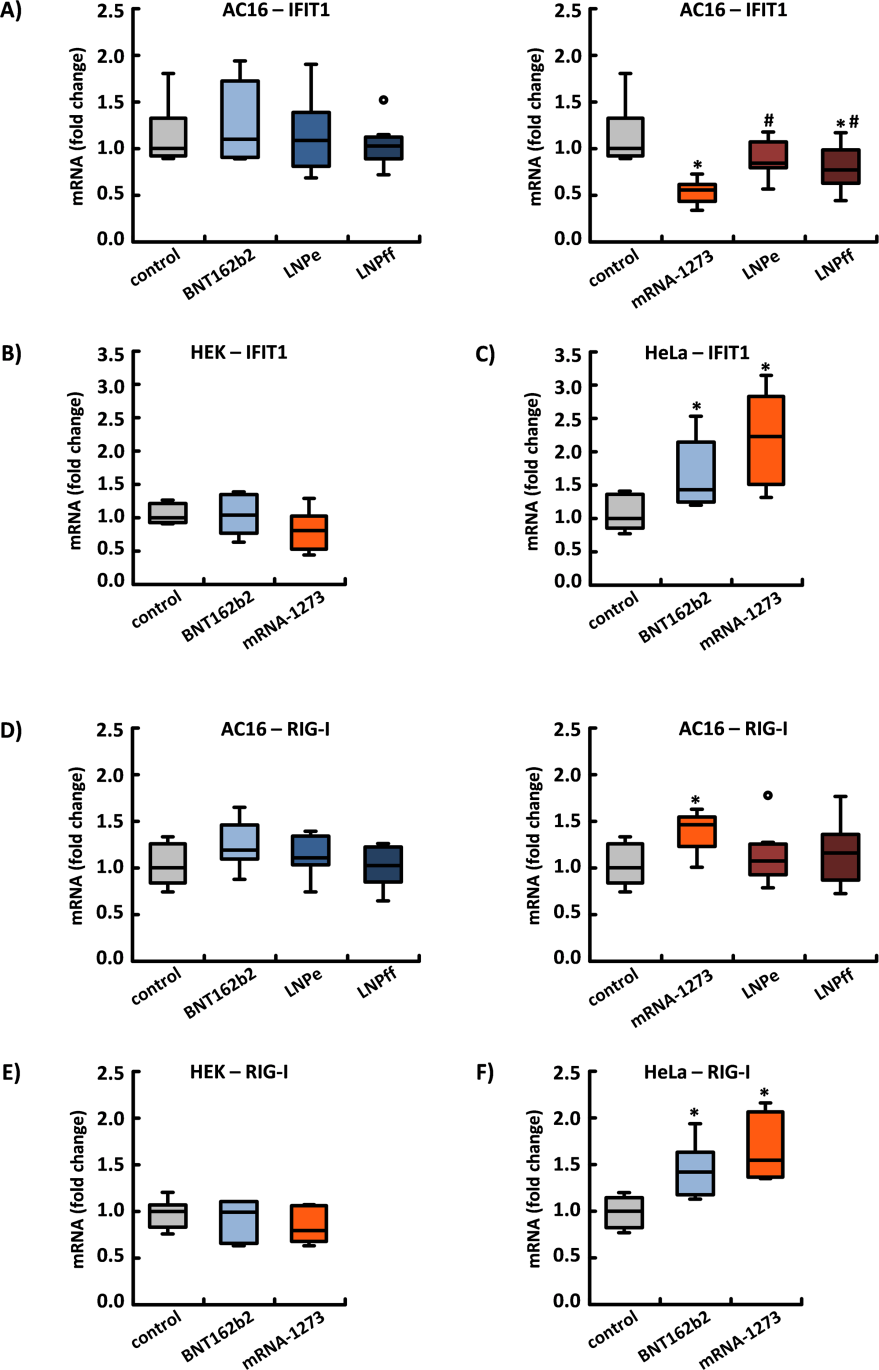
The influence of BNT162b2 and mRNA-1273 on the expression of the RNA-binding proteins IFIT1 and RIG-I. The expression of IFIT1 – the main function of which is to inhibit the translation of viral RNA and accelerate its degradation – was suppressed by mRNA-1273 in **(A)** cardiac AC16 cells and **(B)** tendentially in HEK-293 cells. Furthermore, in AC16 cells, LNPs loaded with Firefly mRNA (LNPff), which are used in mRNA-1273, reduced the expression of IFIT1, while unloaded LNPs (LNPe) did not. In **(C)** HeLa cells, both BNT162b2 and mRNA-1273 induced IFIT1 expression. The expression of RIG-I, which as a cytosolic receptor of the innate immune system can mediate the induction of a type I IFN response, was significantly induced in **(D)** cardiac AC16 cells exclusively by mRNA-1273. By contrast, in **(E)** HEK-293 cells, neither BNT162b2 nor mRNA-1273 had any effect on RIG-I expression. In **(F)** HeLa cells, the application of both mRNAs led to a significant increase in RIG-1 expression. These results are based on n=8 **(A, D)** and n=6 **(B, C, E, F)** culture dishes from n=4 and n=3 cell passages, respectively. *p < 0.05 *vs*. control, #p < 0.05 *vs*. mRNA-1273.

In AC16 cardiomyocytes, neither the application of BNT162b2 nor the associated LNPs had any effect on the mRNA expression of IFIT1 after 24 h. By contrast, mRNA-1273 reduced IFIT1 expression to 56% (0.56 ± 0.11). Unloaded LNPs had no effect on IFIT1 expression, but LNPs loaded with firefly mRNA used in mRNA-1273 reduced IFIT1 expression to 77% (0.77 ± 0.22) (see **Figure 6A**).

In HEK-293 cells, BNT162b2 again had no effect on the expression of IFIT1, while mRNA-1273 again reduced its expression to 81% (0.81 ± 0.28) (not statistically significant). Only in HeLa cells did both BNT162b2 and mRNA-1273 induce increased expression of IFIT1 by 43% (1.43 ± 0.48) and 123% (2.23 ± 0.66), respectively (see **Figures 6B, C**).

In addition, we determined the expression of RIG-I, a cytosolic receptor of the innate immune system that can mediate the induction of a type I IFN response. As an ATP-dependent DExD/H box RNA helicase, RIG-I is both activated and increasingly transcribed by specific recognition features of viral RNA (30).

In AC16 cardiomyocytes, only mRNA-1273 induced the increased expression of RIG-I by 46% (1.46 ± 0.20), respectively, but not the respective LNPs. In HEK-293 cells, RIG-I expression was not affected by either mRNA; by contrast, in HeLa cells, BNT162b2 induced increased expression by 42% (1.42 ± 0.26) and mRNA-1273 by 55% (1.55 ± 0.32) (see **Figures 6D–F**).

### The influence of BNT162b2 and mRNA-1273 on superoxide production

The spike protein of the SARS-CoV-2 virus has already been shown to directly induce the production of ROS in different cell types. We investigated the influence of BNT162b2 and mRNA-1273 on the degree of oxidative stress in AC16, HEK-293, and HeLa cells using the superoxide indicators DHE and MitoSOX.

**Figure 7:**
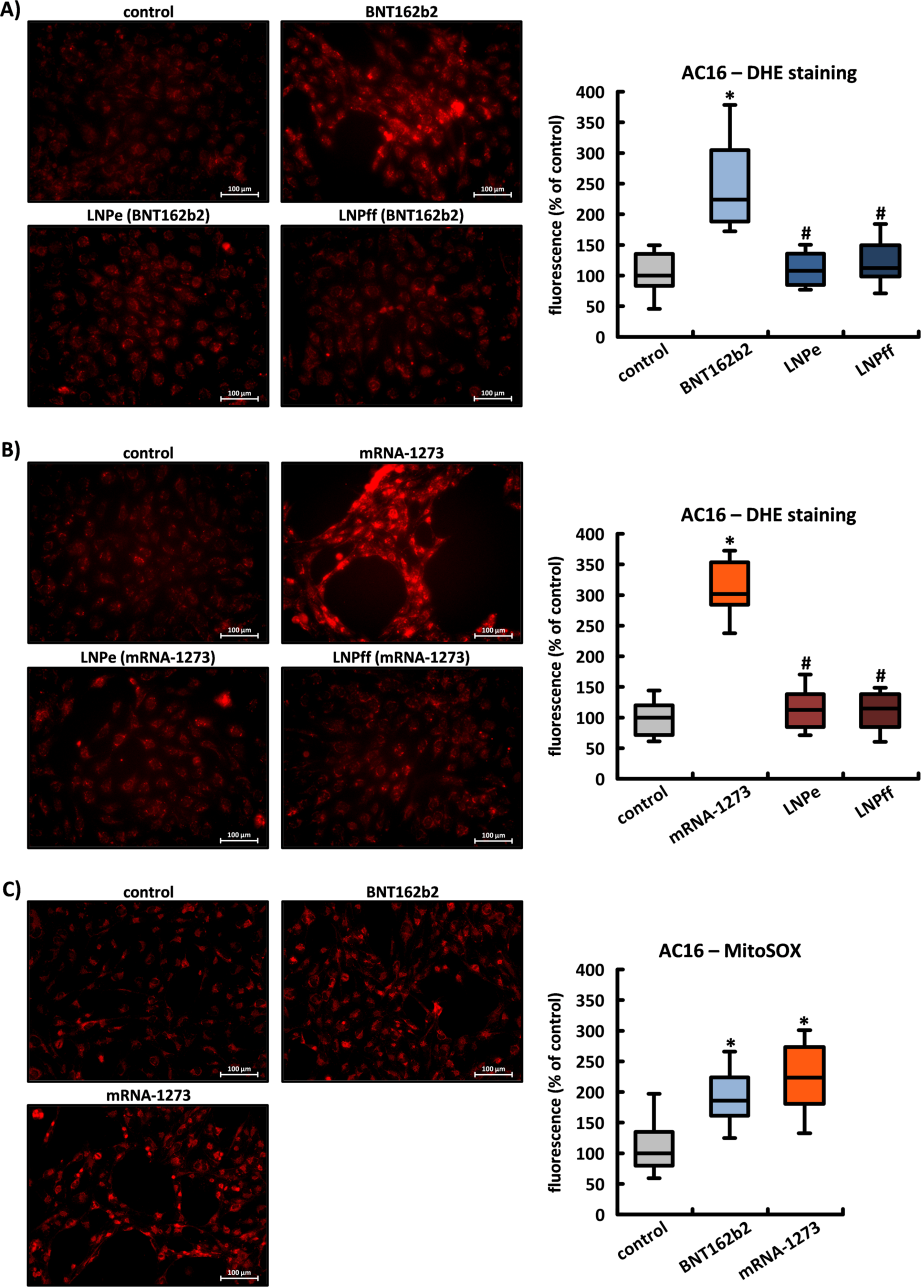
The influence of BNT162b2 and mRNA-1273 on superoxide production. Fluorescence microscopic images of cardiac AC16 cells whose degree of oxidative stress was measured using two superoxide indicators: DHE **(A, B)** and MitoSOX **(C)**. Reactive oxygen species were always detected at 24 h in untreated control cells and after application of BNT162b2 or mRNA-1273 and the corresponding LNPs (LNPe = unloaded; LNPff = loaded with mRNA for Firefly Luciferase). These results are based on n=5 culture dishes from n=5 cell passages; three image sections per culture dish were recorded densitometrically. *p < 0.05 *vs*. control, #p < 0.05 *vs*. BNT162b2 or mRNA-1273.

Microscopic images of DHE staining showed a 2.24x (± 0.61) and 3.02x (± 0.40) increase in relative fluorescence in AC16 cells 24 h after application of BNT162b2 and mRNA-1273, respectively. Neither unloaded nor firefly mRNA-loaded LNPs affected the intensity of the DHE signal. After incubation of the cells with MitoSOX, whose properties specifically allow the detection of ROS in mitochondria, the signal strength increased by a factor of 1.86 (± 0.39) and 2.24 (± 0.50), respectively (see **Figures 7A–C**).

In HEK cells, only BNT162b2 induced the production of superoxide within 24 h, which could be detected by both DHE and MitoSOX (see **Supplementary Figures S1A, B**). In HeLa cells, neither BNT162b2 nor mRNA-1273 influenced the signal strength of either indicator during the same period (see **Supplementary Figures S2A, B**).

### Effects of spike production on the cell morphology of AC16, HEK-293, and HeLa cells

**Figure 8:**
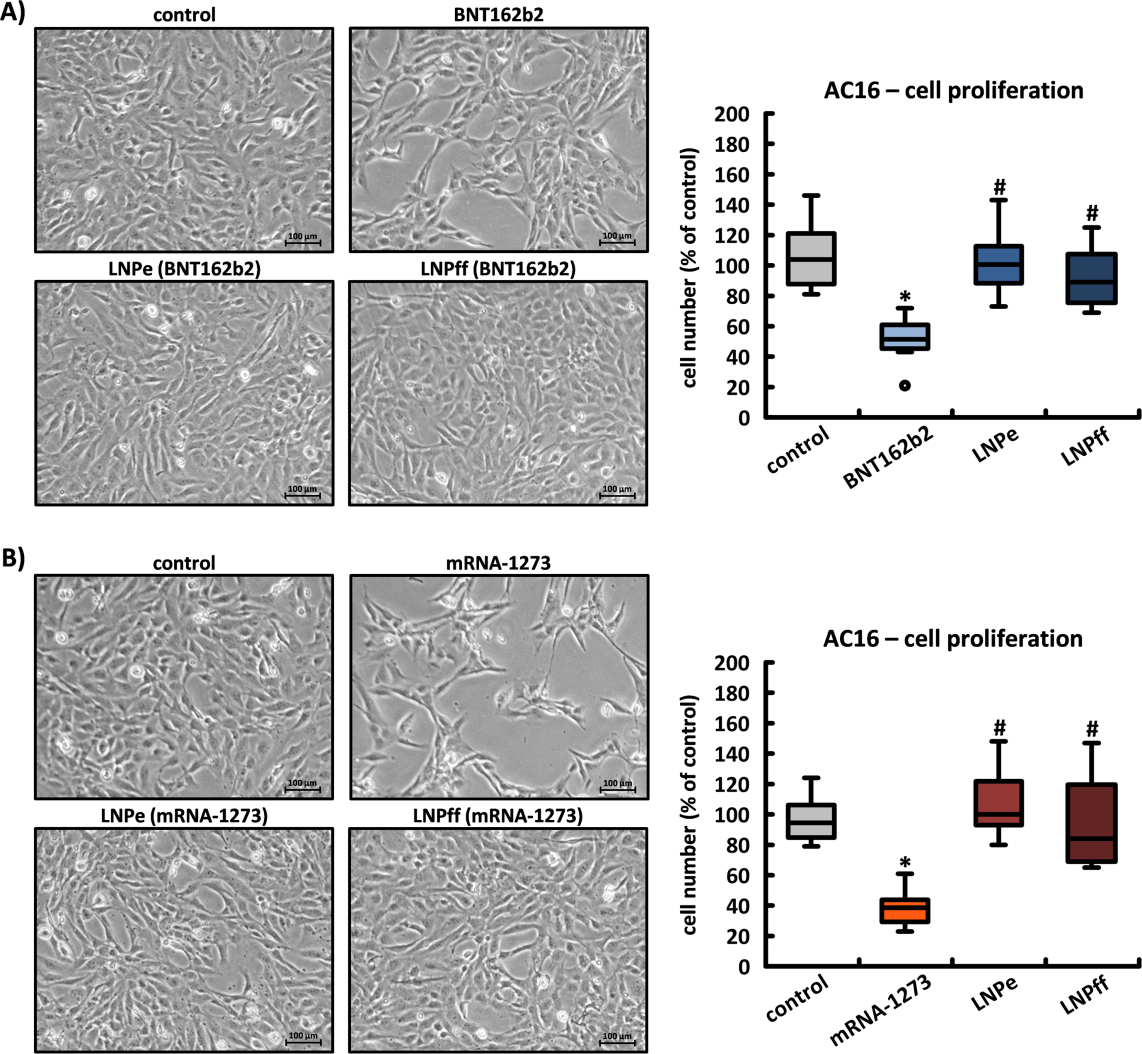
The effects of BNT162b2 and mRNA-1273 on the proliferation of cardiac AC16 cells. An Olympus CKX41 was used to take photographs at 24 h of untreated control cells and cells after application of **(A)** BNT162b2 and **(B)**, mRNA-1273 and the corresponding LNPs (LNPe = unloaded; LNPff = loaded with mRNA for Firefly Luciferase). The cells in two identically sized image sections per culture dish were counted using BZ Advanced Analysis Software (Keyence Corporation, version 3.60). The results are based in each case on n=5 culture dishes from n=5 cell passages. *p < 0.05 *vs*. control, #p < 0.05 *vs*. BNT162b2 or mRNA-1273.

The intracellular production of the spike protein, but not the LNPs or RNA per se, had dramatic effects on cell morphology in AC16 cultures, which was particularly impressive within the first 24 h at relatively low cell density; with an approximately 36 h delay compared to untreated controls, only BNT162b2 incubated cells reached 100% confluence. Corresponding to the higher synthesis rate of the spike protein and its aggregates, mRNA-1273 showed a significantly stronger expression of the described characteristics. Unloaded and firefly mRNA-loaded LNPs did not produce morphological abnormalities or effect cell division in AC16 cardiomyocytes (see **Figures 8A, B**).

In HeLa cells, both BNT162b2 and mRNA-1273 caused a moderate reduction in the proliferation rate; in HEK-293 cell cultures, there was no effect on either morphology or proliferation (see **Supplementary Figures S3A, B**). While HEK-293 cells are known for their high transfection tolerance and robust protein turnover, HeLa cells may exhibit a lower threshold for proteotoxic stress, rendering them more susceptible to the intracellular accumulation of spike-derived aggregates despite lower expression levels.

### Secretion of the spike protein as a prerequisite for the immune response

**Figure 9:**
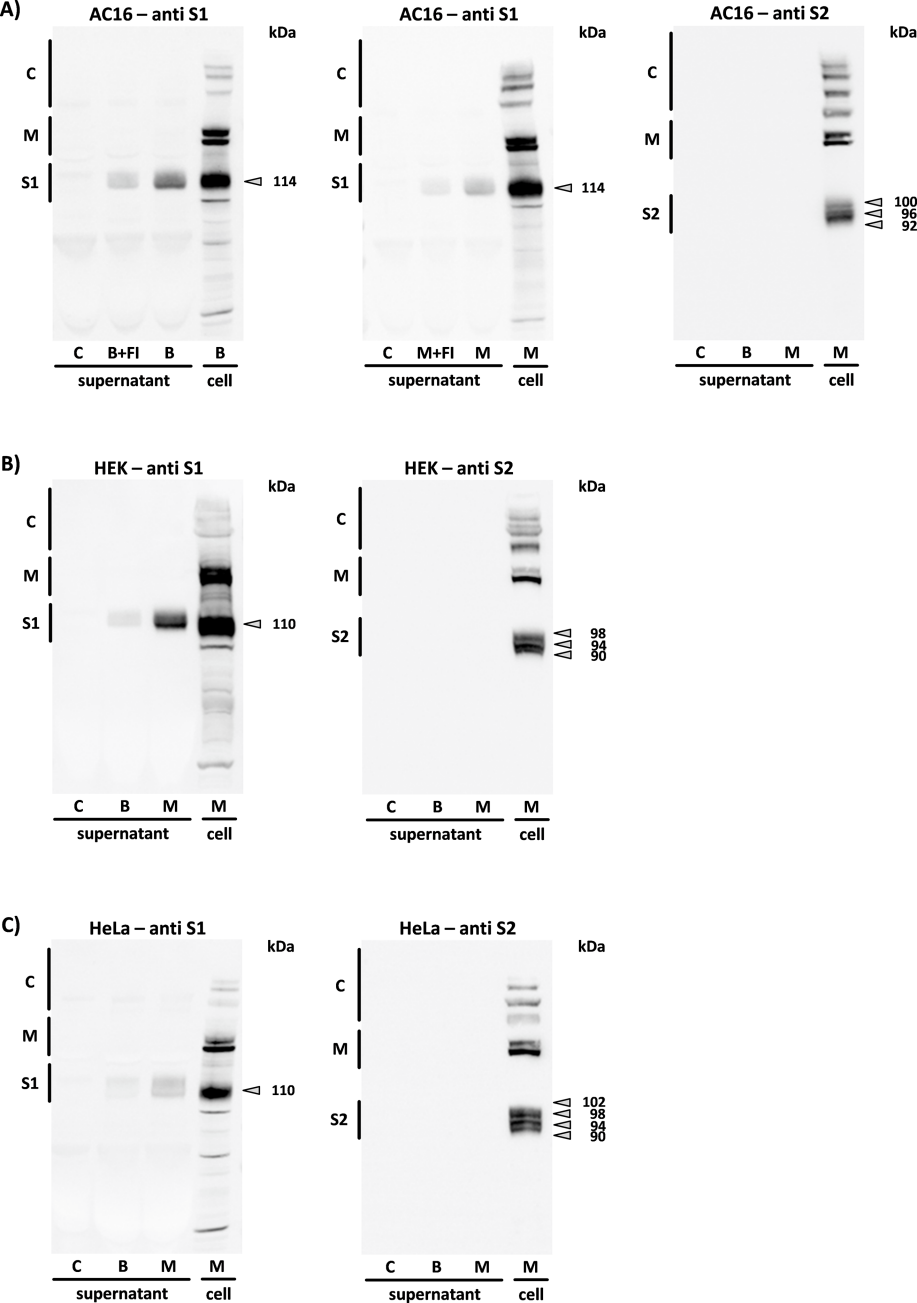
The secretion of the spike (S) subunits. The figure shows all spike protein associated products detected through the S1 or S2 antibodies 24 h after application of BNT162b2 (B), mRNA-1273 (M) and after pre-incubation of the cells with furin inhibitor I (B+FI, M+FI) in the supernatant of **(A)** AC16 cardiomyocytes, **(B)** HEK-293, and **(C)** HeLa cells. The band pattern was reproduced on cells from n=4 cell passages. C = supernatant of untreated controls, cell = reference sample of the respective cell lysate. (S1, S1 subunit; S2, S2 subunit; M, spike monomers; C, complexes consisting of spike monomers and its subunits).

The “supernatants” of all three cell lines were analyzed after 24 h of incubation for secretion products induced by BNT162b2 and mRNA-1273 after translation of the spike monomers. The intracellular band pattern of each cell type was compared with that of the corresponding supernatant. Of the numerous spike monomer derivatives that could be detected in the cell lysate, only the S1 subunit could be detected in the supernatant of all three cell lines (in some cases as double bands). Accordingly, the secretion of the S1 subunit from AC16 cells pre-incubated with furin inhibitor I was reduced compared to untreated cells. It should be noted that the S1 subunit of the supernatant is represented by only 15 µl of the total 5 ml cell culture medium per dish; contrary to the relatively low band intensity, an efficient secretion performance must therefore be assumed (see **Figures 9A–C**).

### The translation of the Omicron-adapted mRNA

Classified by the WHO as a “new variant of concern” in November 2021, B.1.1.529 differed from the original Wuhan variant by an unusually high number of around 30 amino acid changes in the spike protein. To protect against this new SARS-CoV-2 variant, which was given the name “Omicron”, a bivalent vaccine was approved for the first time. The mRNAs of this vaccine encode both the spike monomer of the Wuhan variant and that of the Omicron sublines BA.4/BA.5. We investigated the translation of the spike monomers as well as the secretion of the S1 subunit after application of the Moderna’s bivalent BA.4/BA.5 vaccine (mRNA-1273 222) in AC16 cells.

**Figure 10:**
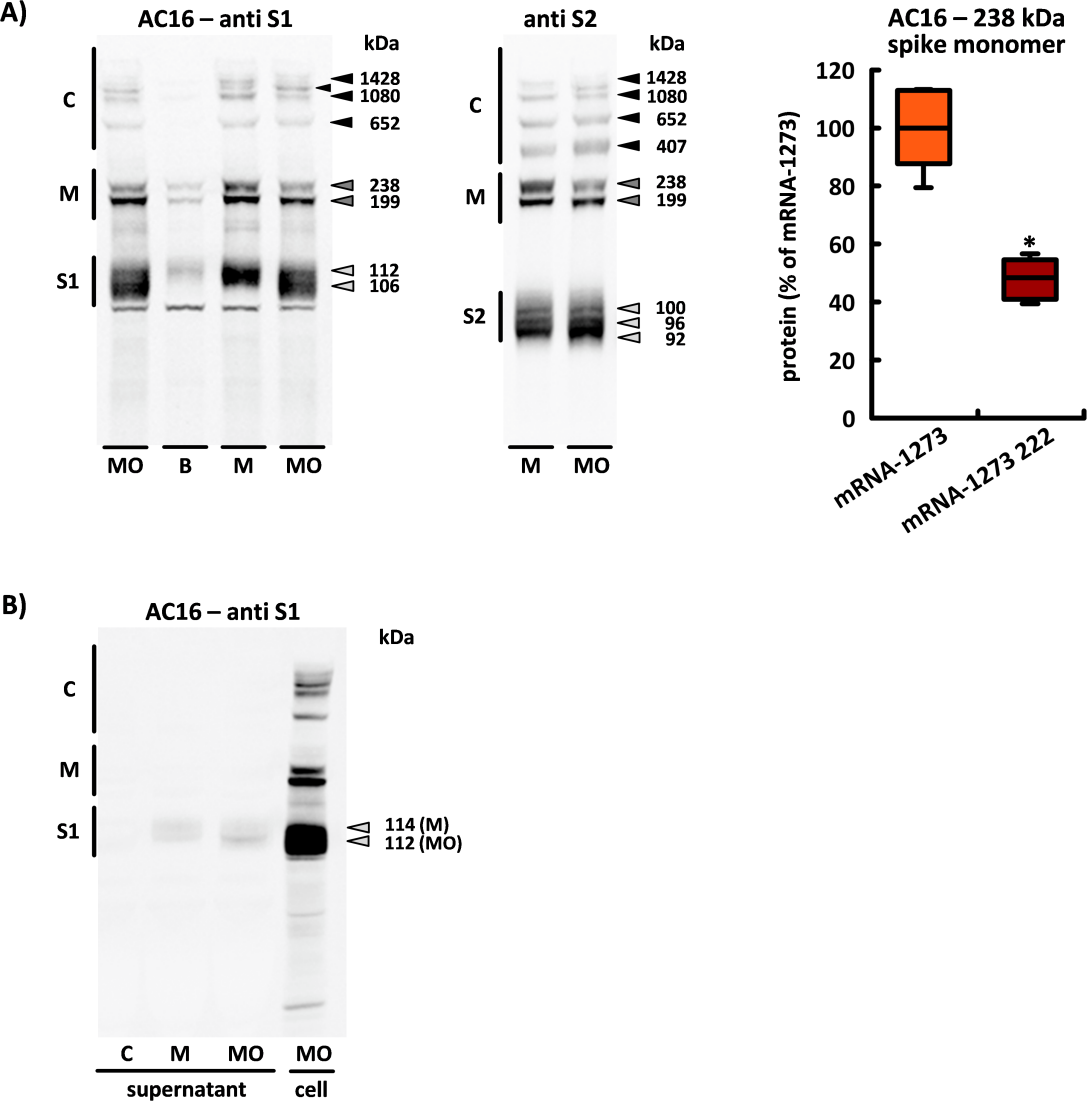
Translation of the bivalent BA.4/BA.5 vaccine mRNA-1273 222.**(A)** This image shows the characteristic band patterns detected through the S1 or S2 antibodies 24 h after application of mRNA-1273 (M), BNT162b2 (B) and mRNA-1273 222 (MO) in AC16 cells. The densitometric results for the 238 kDa spike monomer are based in each case on n=5 culture dishes from n=5 cell passages. **(B)** All spike protein associated products detected by the S1 antibody in the supernatant of AC16 cardiomyocytes within 24 h after application of mRNA-1273 (M) or mRNA-1273 222 (MO). The band pattern was reproduced in each case on cells from n=5 cell passages. C = supernatant of untreated controls, cell = reference sample of the respective cell lysate. *, p< 0.05 *vs*. mRNA-1273. (S1, S1 subunit; S2, S2 subunit; M, spike monomers; C, complexes consisting of spike monomers and its subunits).

The bivalent vaccine also produced two spike monomers with identical molecular weight, but the higher molecular weight product showed a significantly reduced intensity (0.48 ± 0.06). In addition, mRNA-1273–222 induced the formation of two differently sized S1 subunits: the higher molecular weight band (which weighed approximately 112 kDa) had a slightly lower molecular weight than the S1 subunit of the monovalent vaccine; a second S1 subunit was detected with a significantly reduced molecular weight of approximately 106 kDa. There were no differences between the three S2 subunits. In the supernatant, however, we found only the higher molecular weight S1 subunit, which also had a lower molecular weight extracellularly compared to the secreted subunit of the monovalent vaccine (see **Figures 10A, B**).

The bivalent vaccine induced high-molecular complexes that did not differ from the monovalent vaccine in terms of number or molecular weight, but their intensity changed in a characteristic way: “complex 1080” and “complex 1428” showed a reduced intensity, while “complex 1286”, which was initially difficult to detect, showed a significantly increased intensity (see **Figure 10A**).

## Discussion

In the interest of identifying the pathomechanisms that underlie the cardiac side effects attributed to mRNA-based corona vaccination, we investigated the post-translational processing of the encoded spike monomers and their influence on cellular and molecular processes following application of BNT162b2 and mRNA-1273 in human AC16 cardiomyocytes as well as, for comparative purposes, in the non-cardiac cell lines HEK-293 and HeLa.

Our analyses demonstrate that the post-translational processing of the encoded spike monomers is considerably more complex than previously assumed. In our experiments, we consistently detected two translation products of different sizes, irrespective of the cell type. This observation may be explained by the presence of two potential initiation sites for translation. This complexity is of particular relevance in the context of mRNA-based vaccines, in which the regulation of translation, processing, and secretion is determined entirely by the properties of the transfected host cells. Our data confirm this pronounced cell type-dependency and indicate that the resulting derivatives, including non-secreted cleavage products and covalently linked complexes, exert functionally relevant effects on morphology, proliferation, and cellular stress responses.

Our main findings contradict the results obtained by Patel et al., who investigated the translation of BNT162b2 in HEK-293 cells and concluded from digitally generated Western blots that a 230 kDa spike monomer and its two subunits are exclusively responsible for the three expected products (31). The use of ‘ProteinSimple’ separation modules in the range between 12–230 kDa, combined with the potential limitations in resolution and sensitivity of the applied detection method, may have contributed to the failure to detect additional off-target products.

At present, we can only speculate as to the stability and half-life of the spike proteins and their high-molecular complexes, as well as to associated effects on the viability and function of different body cells, with particular attention to irreversible postmitotic cell types such as neurons and cardiomyocytes, which may warrant closer investigation due to their limited regenerative capacity. Neuropathological studies in SARS-CoV-2 patients have documented glial activation, neuroinflammation, and microvascular changes, which may contribute to persistent neurological symptoms long after the acute phase of infection has resolved (32–34). Whether intracellularly expressed spike proteins can contribute to these mechanisms remains unknown, but deserves further study, particularly in the context of irreversible post-mitotic cell types. Supporting this notion, spike-derived high-molecular-weight aggregates were also detected in U343 cells, which exhibit astrocytic properties (see **Supplementary Figure S4**).

The efficiency of spike monomer production varied in relation to the cell type and mRNA applied. After 24 h of incubation, the amount of mRNA-1273 incorporated into AC16 cells was about 10x higher than that of BNT162b2 (this comparison includes adjustments made to account for the 3.3x higher RNA concentration of mRNA-1273). This difference may be attributable to variation in the efficiency of transfection, which, depending on the cell type, is based on the composition of the LNPs used. In addition, both mRNA-1273 and BNT162b2 influence the expression of the RNA-binding protein IFIT1, the downregulation of which can significantly increase both the efficiency of translation and the stability and half-life of an “exogenous” RNA (28, 29). In contrast to BNT162b2, the application of mRNA-1273 to AC16 cardiomyocytes led to a significant reduction in IFIT1 expression, which could explain both the higher mRNA concentration and the more efficient translation of mRNA-1273. In the case of HeLa cells, by contrast, IFIT1 expression was significantly induced regardless of the mRNA applied. HeLa cells also had the lowest intracellular concentration of mRNA and produced comparatively fewer spike monomers. Of the three cell types considered in our experiments, cardiac AC16 cells showed both the highest number of incorporated mRNA copies and the most efficient translation of the encoded spike protein.

The biodistribution of LNP-based mRNA vaccines has been investigated in multiple preclinical and clinical studies. The Pfizer report (SARS-CoV-2 mRNA Vaccine, Pharmacokinetics: Organ Distribution Continued, Report No. 185350, 7) demonstrated LNP uptake in several organs, including the heart. Complementary evidence from recent reviews and human studies indicates that LNP-delivered mRNA can enter the circulation following intramuscular administration and may subsequently be taken up by peripheral tissues (8–11). Together, these findings support the notion that transfection of cells distant from the injection site is plausible. Furthermore, a case report by Yamamoto et al. demonstrated persistent detection of the encoded spike protein in a cutaneous varicella-zoster lesion several weeks after mRNA vaccination, highlighting the potential for prolonged intracellular expression in certain tissues (35).

In the study by Krauson et al., SARS-CoV-2 vaccine mRNA was detected in the myocardium of three out of twenty patients who died within 30 days of mRNA vaccination. These patients exhibited signs of healing myocardial injury, characterized by increased macrophage infiltration, whereas no histological myocarditis was identified. Although direct evidence of spike protein expression was not achieved, the findings indicate that vaccine-derived mRNA can persist in cardiac tissue under specific pathological conditions (36). This observation is consistent with previous reports demonstrating that cardiac muscle tissue can serve as a receptive target for LNP-delivered mRNA (37, 38). However, the extent and duration of expression are likely modulated by local RNase activity, intracellular trafficking, and the immunological milieu (potentially including IFIT1, as supported by our own data) thereby influencing RNA stability and translation efficiency in a cell type-specific manner (39, 40).

Although quantitative data on myocardial uptake, biodistribution, and spike protein expression following mRNA vaccination in humans remain limited, our findings suggest that even low concentrations of LNPs reaching the myocardium may be sufficient to elicit myocarditis, which has been histopathologically classified as atypical following COVID-19 mRNA vaccination (41). In an autopsy-based study of twenty-five patients who unexpectedly died within twenty days of mRNA vaccination, Schwab et al. identified focal interstitial T-lymphocytic infiltration in the myocardium associated with mild myocyte damage in four cases. Collectively, these autopsy findings indicated death due to acute arrhythmogenic cardiac events (42).

Given the unique electrophysiological and structural properties of cardiac tissue, even small focal lesions within the myocardium can exert disproportionately significant functional effects. Such lesions may disrupt electrical conduction or alter cardiac neural innervation, potentially leading to subclinical or clinical manifestations (43–45).

While the risk of cardiovascular complications following SARS-CoV-2 infection remains significantly higher in the general population, the rare incidence of myocarditis following mRNA-based COVID-19 vaccination continues to merit close attention. This view is supported by the analysis of Sharff et al., which identifies methodological limitations in the recording of vaccine-associated myocarditis cases (18), and in particular by the follow-up data from the MACiV multicenter study (46). This study analyzed a cohort of 333 young people with mRNA-induced myocarditis and showed that 60% of the 161 patients with initial late gadolinium enhancement (LGE) showed persistent myocardial changes even after a median follow-up period of 159 days. These observations emphasize the need for a differentiated risk/benefit assessment, particularly in younger target groups, as well as careful follow-up of mild cases.

Our findings on the intracellular processing of the encoded spike protein, together with the subsequent cellular effects discussed below, provide a scientifically substantiated explanation for the clinically observed cardiovascular side effects associated with mRNA-based SARS-CoV-2 vaccination. However, we must explicitly emphasize that the extrapolation of these results to the whole organism – particularly with respect to the locally achievable exposure levels in peripheral tissues – requires clinical validation in future translational studies. Moreover, forthcoming investigations should systematically assess the extent to which the observed effects occur in a dose- and cell type-dependent manner, and define the concentration thresholds at which comparable, albeit attenuated, cellular stress responses are elicited.

In their work, Buoninfante et al. summarize the possible causes of the cardiac inflammation induced by mRNA-based corona vaccination. They conclude that no clear pathomechanism has been identified to date, and that further epidemiological, clinical, and non-clinical research is needed to determine the underlying causal mechanisms (47). Our findings suggest that the observed adverse reactions are an inflammatory response to the spike protein-related products that are formed within the cell. Both BNT162b2 and mRNA-1273 had no effect on IL-6 expression in HEK-293 cells, but caused a moderate induction in HeLa cells. In cardiac AC16 cells, both mRNAs had the strongest effect on the expression of IL-6. However, mRNA-1273 induced an approximately 9x stronger increase, which can be attributed to both the higher amount of spike proteins produced and the composition of the selected LNPs (which cause a significant increase in IL-6 expression, even in an unloaded state) (48–51).

Our experiments demonstrated serious cell type-specific differences in the inflammatory risk profile, and also identified the comparatively high risk potential to which myocardial cells are exposed. These findings are significant because of their link to both the long-term prognosis of myocardial inflammation and the importance of IL-6-mediated signaling mechanisms, which have recently been identified as therapeutic targets, due to their association with the pathogenesis and progression of heart failure (52, 53).

In their studies, which rely on cell-free systems, both Petruk et al. and Petrlova et al. demonstrate that SARS-CoV-2 spike protein can form high-molecular weight aggregates in the presence of LPS. In cultures of immunocompetent cells and *in vivo* in the mouse model, pro-inflammatory signaling pathways were activated only upon formation of aggregated spike protein complexes (54, 55). It is therefore plausible that the IL-6 induction observed in AC16 and HeLa cells may also be driven by high-molecular-weight off-target products rather than the spike monomer itself.

Induced by viral infection, the RNA receptor RIG-I triggers important type I IFN-mediated immune reactions. In the heart muscle, however, maladaptive effects are predominantly ascribed to type I IFNs, given the absence of a viral infection (56, 57). In our experiments, the expression of RIG-I was more strongly induced in HeLa cells (which primarily have epithelial properties) than in cardiac AC16 cells. In the vascular system, type I IFNs cause endothelial dysfunction, which is thought to be responsible for various pathologies, including accelerated thrombosis, due to increased platelet activation (58). Future studies will need to clarify the influence of mRNA technology on proteins whose triggered immune reactions normally serve to defend against RNA viruses.

In recent years, several research groups have demonstrated the induction of oxidative stress in various cells as a direct consequence of the application or plasmid-based expression of spike proteins (59–61). Numerous findings indicate that infection with SARS-CoV-2 directly impairs mitochondrial function and induces the production of ROS. The interaction of the spike protein with endogenous proteins is thought to be the predominant cause of the observed mitochondrial pathophysiology (62). Our results have confirmed that the vaccine-encoded spike proteins can induce the production of superoxide involving the mitochondria. However, this effect was subject to large differences specific to each cell type. These differences could be attributable to the concentration of produced spike monomers or to the specific post-translational processing. After 24 h of incubation, both superoxide indicators (DHE, MitoSOX) revealed no sign of oxidative stress in HeLa cells. In HEK-293 cells, oxidative stress was only registered following application of BNT162b2. The massive induction in AC16 cardiomyocytes must be taken into account, as oxidative stress in the heart muscle has a negative effect on calcium balance and contractile function, and can also trigger arrhythmias and maladaptive remodeling (63, 64).

There was a significant and comparatively higher increase in the relative fluorescence of the non-specific superoxide indicator DHE, indicating that mitochondria are most likely not the only sources of ROS activated by intracellular spike proteins.

In all three cell lines, only the S1 subunit could be detected in the supernatant. The immune response is therefore most likely primarily directed against the subunit whose N-terminus contains the receptor-binding domain (RBD), whereas the membrane-anchored S2 subunit may still contribute to antigen presentation and cellular immune activation. However, our results are consistent with the findings of Wheeler et al. who, after mRNA-based corona vaccinations, almost exclusively detected antibodies directed against epitopes of the RBD or the S1 subunit, but not against the S2 subunit (65). According to Heo et al., vaccines whose neutralizing antibodies are directed at the relatively conserved S2 subunit could play an important role as “universal” vaccines to combat numerous SARS-CoV-2 variants (66).

Furin, an endoprotease of the proprotein convertase family, primarily catalyzes the proteolytic maturation of precursor proteins in the eukaryotic protein secretion pathway in the trans-Golgi network (TGN) (67). After translation of the encoded spike monomers and their uptake into the endoplasmic reticulum (ER), the two S subunits are proteolytically formed in the Golgi complex (GC) or in the TGN, from which only the S1 subunit is secreted. The exact mechanisms by which protein fate is decided in the TGN are still largely unknown (68). This fact is also reflected by our findings regarding bivalent BA.4/BA.5 adapted mRNA, which induced the formation of two S1 subunits in AC16 cardiomyocytes, but only one of which could be detected in the supernatant.

As proteins travel from the ER to the TGN, they undergo meticulous processing to ensure proper synthesis, accurate folding, and flawless assembly. Various causes for the formation of protein aggregates in the ER and GC have been identified. Failure to efficiently remove covalently bound spike protein complexes can result in their accumulation in the ER, leading to toxic protein aggregates that can cause cell and tissue damage (69, 70).

Our findings collectively indicate that the intracellular accumulation of various spike protein derivatives may lead to potentially proteotoxic consequences. Notably, pretreatment with a furin inhibitor suppresses the formation of larger S1/S2 complexes but simultaneously promotes the aggregation of unprocessed monomers (see **Figure 2**). In AC16 cardiomypcytes, these alterations are associated, as shown in **Supplementary Figure S5**, with elevated mitochondrial superoxide levels and persistently reduced cell proliferation.

After 24 h, a complex cellular picture emerges: despite furin inhibition, both unprocessed spike monomers and (delayed) cleaved fragments remain detectable. Both forms display aggregation behavior and contribute to cellular stress responses. The observed increases in ROS levels and morphological deterioration likely reflect a combined effect of these protein species. Although furin inhibition precluded the detection of IL-6 upregulation, most likely due to the well-documented anti-inflammatory effects of DMSO in various cell models (71, 72), prior experiments without DMSO had consistently shown IL-6 induction. Interestingly, however, in the presence of furin inhibitor, mRNA-1273 still caused a moderate but statistically significant increase in IL-6 expression compared to the DMSO control, suggesting a more robust proinflammatory potential than BNT162b2 even under anti-inflammatory conditions.

This suggests that intracellular accumulation of spike proteins may activate inflammatory pathways, potentially via ER stress and the unfolded protein response (UPR). The persistence of oxidative stress and impaired proliferation despite suppression of IL-6 expression indicates that these stress responses are not necessarily coupled, but may proceed through partially independent pathways. This uncoupling effect, likely involving redox-sensitive inhibition of transcriptional regulators such as NF-κB or STAT3, highlights the distinct regulatory layers of oxidative and inflammatory signaling (73).

Although AC16 cells do not replicate the contractile behavior of adult cardiomyocytes, they retain key metabolic and structural features of cardiac tissue, including oxidative mitochondrial function and expression of cardiac-specific stress markers, making them a suitable *in vitro* model for investigating proteotoxicity and mitochondrial responses in a human cardiac context (24, 74, 75).

Current evidence suggests that effects induced by the mRNA-based COVID-19 vaccines may potentially explain the numerous symptoms associated with post-Covid syndromes (also known as “long Covid”), as it is highly likely that the spike protein of the coronavirus undergoes post-translational processing in a manner similar to the spike proteins produced by the two mRNA vaccines. This assumption would also explain why Nuvaxovid, a conventional vaccine, has a markedly better side-effect profile, as it involves the direct administration of an already prepared prefusion-stabilized spike protein, bypassing the need for cellular production in the body (76). In contrast to the intracellular translation and processing of mRNA-derived spike proteins, which can result in off-target effects, intracellular accumulation, and aggregation, the protein-based approach of Nuvaxovid avoids these risks entirely. Our findings underscore that it is not merely the spike protein itself, but its intracellular production and post-translational handling, that contribute to the distinct cellular stress responses observed following mRNA vaccination.

## Summary and conclusion

The risk profile of mRNA-induced immunization cannot be generally defined at present, as the non-cell type-specific LNPs currently in use may reach distant cells following injection, and the nature and extent of potential side effects depend on the transfected cell types and the properties of the immunogen. Given the exceptionally high safety standards required for vaccines, a precise understanding of the intracellular processing of encoded proteins and their potential impact on cellular physiological parameters is essential for mRNA-based applications before they advance to clinical use.

The two mRNA-based corona vaccines translate their encoded spike monomers in human cells with varying degrees of efficiency, from which one on-target and numerous off-target products develop within a short time frame. Among the three cell lines we examined, AC16 cardiomyocytes exhibited a particularly unfavorable risk profile, as they developed both an inflammatory phenotype and oxidative stress as a result of the post-translational formation of spike protein derivatives.

Our findings underscore that ensuring a robust cardiac safety profile of mRNA-based therapeutics requires careful preclinical evaluation of intracellular protein processing, off-target products, and cell type-specific stress responses. These mechanistic insights may help guide the rational design and future optimization of next-generation mRNA platform technologies.

## Data Availability

The raw data supporting the conclusions of this article will be made available by the authors, without undue reservation.

## References

[1] Hielscher F Schmidt T Klemis V Wilhelm A Marx S Abu-Omar A . NVX-CoV2373-induced cellular and humoral immunity towards parental SARS-CoV-2 and VOCs compared to BNT162b2 and mRNA-1273-regimens. J Clin Virol. (2022) 157:105321. doi: 10.1016/j.jcv.2022.105321, PMID: 36279695 PMC9576915

[2] Bennett C Rivers EJ Woo W Bloch M Cheung K Griffin P . Immunogenicity and safety of heterologous Omicron BA.1 and bivalent SARS-COV-2 recombinant spike protein booster vaccines: a Phase 3 randomized clinical trial. J Infect Dis. (2023) 230:e4–e16. doi: 10.1093/infdis/jiad508, PMID: 39052718 PMC11272042

[3] Heath PT Galiza EP Baxter DN Boffito M Browne D Burns F . Safety and efficacy of the NVX-COV2373 Coronavirus Disease 2019 vaccine at completion of the Placebo-Controlled Phase of a randomized controlled trial. Clin Infect Dis. (2022) 76:398–407. doi: 10.1093/cid/ciac803, PMID: 36210481 PMC9619635

[4] Teo SP . Review of COVID-19 mRNA vaccines: BNT162b2 and mRNA-1273. J Pharm Pract. (2021) 35:947–51. doi: 10.1177/08971900211009650, PMID: 33840294

[5] Hassett KJ Rajlic IL Bahl K White R Cowens K Jacquinet E . mRNA vaccine trafficking and resulting protein expression after intramuscular administration. Mol Ther Nucleic Acids. (2023) 35:102083. doi: 10.1016/j.omtn.2023.102083, PMID: 38161733 PMC10755037

[6] Kim S Jeon JH Kim M Lee Y Hwang Y-H Park M . Innate immune responses against mRNA vaccine promote cellular immunity through IFN-β at the injection site. Nat Commun. (2024) 15:7226. doi: 10.1038/s41467-024-51411-9, PMID: 39191748 PMC11349762

[7] SARS-CoV-2 mRNA Vaccine (BNT162, PF-0 7302048): 2.6.5.5B. Pharmacokinetics: organ distribution continued, report number: 185350. Available online at: https://www.pmda.go.jp/drugs/2021/P20210212001/672212000_30300AMX00231_I100_2.pdf. (Accessed December 2024).

[8] Pateev I Seregina K Ivanov R Reshetnikov V . Biodistribution of RNA vaccines and of their products: evidence from human and animal studies. Biomedicines. (2023) 12:59. doi: 10.3390/biomedicines12010059, PMID: 38255166 PMC10812935

[9] Takanashi A Pouton CW Al-Wassiti H . Delivery and Expression of mRNA in the Secondary Lymphoid Organs Drive Immune Responses to Lipid Nanoparticle-mRNA Vaccines after Intramuscular Injection. Mol Pharm. (2023) 20:3876–85. doi: 10.1021/acs.molpharmaceut.2c01024, PMID: 37491979 PMC10411422

[10] Kent SJ Li S Amarasena TH Reynaldi A Lee WS Leeming MG . Blood distribution of SARS-COV-2 lipid nanoparticle mRNA vaccine in humans. ACS Nano. (2024) 18:27077–89. doi: 10.1021/acsnano.4c11652, PMID: 39298422 PMC11447892

[11] Castruita JAS Schneider UV Mollerup S Leineweber TD Weis N Bukh J . SARS-CoV-2 spike mRNA vaccine sequences circulate in blood up to 28 days after COVID-19 vaccination. Apmis. (2023) 131:128–32. doi: 10.1111/apm.13294, PMID: 36647776 PMC10107710

[12] D’Angelo T Cattafi A Carerj ML Booz C Ascenti G Cicero G . Myocarditis after SARS-COV-2 vaccination: A Vaccine-Induced Reaction? Can J Cardiol. (2021) 37:1665–7. doi: 10.1016/j.cjca.2021.05.010, PMID: 34118375 PMC8187737

[13] Heymans S Cooper LT . Myocarditis after COVID-19 mRNA vaccination: clinical observations and potential mechanisms. Nat Rev Cardiol. (2021) 19:75–7. doi: 10.1038/s41569-021-00662-w, PMID: 34887571 PMC8656440

[14] Buchan SA Seo CY Johnson C Alley S Kwong JC Nasreen S . Epidemiology of myocarditis and pericarditis following mRNA vaccination by vaccine product, schedule, and interdose interval among adolescents and adults in Ontario, Canada. JAMA Netw Open. (2022) 5:e2218505. doi: 10.1001/jamanetworkopen.2022.18505, PMID: 35749115 PMC9233237

[15] Chua GT Kwan MYW Chui CSL Smith RD Cheung ECL Ma T . Epidemiology of Acute Myocarditis/Pericarditis in Hong Kong adolescents following comirnaty vaccination. Clin Infect Dis. (2021) 75:673–81. doi: 10.1093/cid/ciab989, PMID: 34849657 PMC8767823

[16] Karlstad Ø Hovi P Husby A Härkänen T Selmer RM Pihlström N . SARS-COV-2 vaccination and myocarditis in a Nordic cohort study of 23 million residents. JAMA Cardiol. (2022) 7:600. doi: 10.1001/jamacardio.2022.0583, PMID: 35442390 PMC9021987

[17] Vu SL Bertrand M Jabagi M-J Botton J Drouin J Baricault B . Age and sex-specific risks of myocarditis and pericarditis following Covid-19 messenger RNA vaccines. Nat Commun. (2022) 13. doi: 10.1038/s41467-022-31401-5, PMID: 35752614 PMC9233673

[18] Sharff KA Dancoes DM Longueil JL Johnson ES Lewis PF . Risk of myopericarditis following COVID-19 mRNA vaccination in a large integrated health system: A comparison of completeness and timeliness of two methods. Pharmacoepidemiol Drug Saf. (2022) 31:921–5. doi: 10.1002/pds.5439, PMID: 35404496 PMC9088632

[19] Ferdinandy P Baczkó I Bencsik P Giricz Z Görbe A Pacher P . Definition of hidden drug cardiotoxicity: paradigm change in cardiac safety testing and its clinical implications. Eur Heart J. (2018) 40:1771–7. doi: 10.1093/eurheartj/ehy365, PMID: 29982507 PMC6554653

[20] Schreckenberg R Woitasky N Itani N Czech L Ferdinandy P Schulz R . Cardiac side effects of RNA-based SARS-CoV-2 vaccines: Hidden cardiotoxic effects of mRNA-1273 and BNT162b2 on ventricular myocyte function and structure. Br J Pharmacol. (2023) 181:345–61. doi: 10.1111/bph.16262, PMID: 37828636

[21] Clemens DJ Ye D Zhou W Kim CSJ Pease DR Navaratnarajah CK . SARS-CoV-2 spike protein-mediated cardiomyocyte fusion may contribute to increased arrhythmic risk in COVID-19. PloS One. (2023) 18:e0282151. doi: 10.1371/journal.pone.0282151, PMID: 36888581 PMC9994677

[22] Yasmin F Najeeb H Naeem U Moeed A Atif AR Asghar MS . Adverse events following COVID-19 mRNA vaccines: A systematic review of cardiovascular complication, thrombosis, and thrombocytopenia. Immun Inflamm Dis. (2023) 11. doi: 10.1002/iid3.807, PMID: 36988252 PMC10022421

[23] Mansanguan S Charunwatthana P Piyaphanee W Dechkhajorn W Poolcharoen A Mansanguan C . Cardiovascular manifestation of the BNT162b2 mRNA COVID-19 vaccine in adolescents. Trop Med Infect Dis. (2022) 7:196. doi: 10.3390/tropicalmed7080196, PMID: 36006288 PMC9414075

[24] Davidson M Nesti C Palenzuela L Walker W Hernandez E Protas L . Novel cell lines derived from adult human ventricular cardiomyocytes. J Mol Cell Cardiol. (2005) 39:133–47. doi: 10.1016/j.yjmcc.2005.03.003, PMID: 15913645

[25] Trepotec Z Geiger J Plank C Aneja MK Rudolph C . Segmented poly(A) tails significantly reduce recombination of plasmid DNA without affecting mRNA translation efficiency or half-life. RNA. (2019) 25:507–18. doi: 10.1261/rna.069286.118, PMID: 30647100 PMC6426288

[26] Aranda PS LaJoie DM Jorcyk CL . Bleach gel: A simple agarose gel for analyzing RNA quality. Electrophoresis. (2012) 33:366–9. doi: 10.1002/elps.201100335, PMID: 22222980 PMC3699176

[27] Livak KJ Schmittgen TD . Analysis of relative gene expression data using Real-Time Quantitative PCR and the 2–ΔΔCT method. Methods. (2001) 25:402–8. doi: 10.1006/meth.2001.1262, PMID: 11846609

[28] Diamond MS Farzan M . The broad-spectrum antiviral functions of IFIT and IFITM proteins. Nat Rev Immunol. (2012) 13:46–57. doi: 10.1038/nri3344, PMID: 23237964 PMC3773942

[29] Geng J Chrabaszczewska M Kurpiejewski K Stankiewicz-Drogon A Jankowska-Anyszka M Darzynkiewicz E . Cap-related modifications of RNA regulate binding to IFIT proteins. RNA. (2024) 30:1292–305. doi: 10.1261/rna.080011.124, PMID: 39009378 PMC11404448

[30] Thoresen DT Galls D Götte B Wang W Pyle AM . A rapid RIG-I signaling relay mediates efficient antiviral response. Mol Cell. (2022) 83:90–104.e4. doi: 10.1016/j.molcel.2022.11.018, PMID: 36521492 PMC9825657

[31] Patel HK Zhang K Utegg R Stephens E Salem S Welch H . Characterization of BNT162B2 mRNA to evaluate risk of Off-Target Antigen Translation. J Pharm Sci. (2023) 112:1364–71. doi: 10.1016/j.xphs.2023.01.007, PMID: 36642376 PMC9836996

[32] Heneka MT Golenbock D Latz E Morgan D Brown R . Immediate and long-term consequences of COVID-19 infections for the development of neurological disease. Alzheimers Res Ther. (2020) 12. doi: 10.1186/s13195-020-00640-3, PMID: 32498691 PMC7271826

[33] Boldrini M Canoll PD Klein RS . How COVID-19 affects the brain. JAMA Psychiatry. (2021) 78:682. doi: 10.1001/jamapsychiatry.2021.0500, PMID: 33769431 PMC9894299

[34] Edinoff AN Chappidi M Alpaugh ES Turbeville BC Falgoust EP Cornett EM . Neurological and psychiatric symptoms of COVID-19: A narrative review. Psychiatry Int. (2022) 3:158–68. doi: 10.3390/psychiatryint3020013

[35] Yamamoto M Kase M Sano H Kamijima R Sano S . Persistent varicella zoster virus infection following mRNA COVID-19 vaccination was associated with the presence of encoded spike protein in the lesion. J Cutan Immunol Allergy. (2022) 6:18–23. doi: 10.1002/cia2.12278

[36] Krauson AJ Casimero FVC Siddiquee Z Stone JR . Duration of SARS-CoV-2 mRNA vaccine persistence and factors associated with cardiac involvement in recently vaccinated patients. NPJ Vaccines. (2023) 8. doi: 10.1038/s41541-023-00742-7, PMID: 37758751 PMC10533894

[37] Chen J Xu Y Zhou M Xu S Varley AJ Golubovic A . Combinatorial design of ionizable lipid nanoparticles for muscle-selective mRNA delivery with minimized off-target effects. Proc Natl Acad Sci USA. (2023) 120. doi: 10.1073/pnas.2309472120, PMID: 38060560 PMC10723144

[38] Żak MM Kaur K Yoo J Kurian AA Adjmi M Mainkar G . Modified mRNA formulation and stability for cardiac and skeletal muscle delivery. Pharmaceutics. (2023) 15:2176. doi: 10.3390/pharmaceutics15092176, PMID: 37765147 PMC10535735

[39] Kornienko IV Aramova O Tishchenko AA Rudoy DV Chikindas ML . RNA stability: A review of the role of structural features and environmental conditions. Molecules. (2024) 29:5978. doi: 10.3390/molecules29245978, PMID: 39770066 PMC11676819

[40] Perlegos AE Byrns CN Bonini NM . Cell type-specific regulation of m6A modified RNAs in the aging Drosophila brain. Aging Cell. (2024) 23. doi: 10.1111/acel.14076, PMID: 38205931 PMC10928574

[41] Gill JR Tashjian R Duncanson E . Autopsy Histopathologic cardiac findings in 2 adolescents following the second COVID-19 vaccine dose. Arch Pathol Lab Med. (2022) 146:925–9. doi: 10.5858/arpa.2021-0435-sa, PMID: 35157759

[42] Schwab C Domke LM Hartmann L Stenzinger A Longerich T Schirmacher P . Autopsy-based histopathological characterization of myocarditis after anti-SARS-CoV-2-vaccination. Clin Res Cardiol. (2022) 112:431–40. doi: 10.1007/s00392-022-02129-5, PMID: 36436002 PMC9702955

[43] Ajijola OA Yagishita D Patel KJ Vaseghi M Zhou W Yamakawa K . Focal myocardial infarction induces global remodeling of cardiac sympathetic innervation: neural remodeling in a spatial context. Am J Physiol Heart Circ Physiol. (2013) 305:H1031–40. doi: 10.1152/ajpheart.00434.2013, PMID: 23893167 PMC3798751

[44] Frustaci A Verardo R Alfarano M Chimenti C . Inflammation of conduction tissue in patients with arrhythmic phenotype of myocarditis. J Clin Med. (2020) 9:3470. doi: 10.3390/jcm9113470, PMID: 33137883 PMC7693374

[45] Tschöpe C Ammirati E Bozkurt B Caforio ALP Cooper LT Felix SB . Myocarditis and inflammatory cardiomyopathy: current evidence and future directions. Nat Rev Cardiol. (2020) 18:169–93. doi: 10.1038/s41569-020-00435-x, PMID: 33046850 PMC7548534

[46] Jain SS Anderson SA Steele JM Wilson HC Muniz JC Soslow JH . Cardiac manifestations and outcomes of COVID-19 vaccine-associated myocarditis in the young in the USA: longitudinal results from the Myocarditis After COVID Vaccination (MACiV) multicenter study. EClinicalMedicine. (2024) 76:102809. doi: 10.1016/j.eclinm.2024.102809, PMID: 39290640 PMC11406334

[47] Buoninfante A Andeweg A Genov G Cavaleri M . Myocarditis associated with COVID-19 vaccination. NPJ Vaccines. (2024) 9. doi: 10.1038/s41541-024-00893-1, PMID: 38942751 PMC11213864

[48] Tsilingiris D Vallianou NG Karampela I Liu J Dalamaga M . Potential implications of lipid nanoparticles in the pathogenesis of myocarditis associated with the use of mRNA vaccines against SARS-CoV-2. Metabol Open. (2021) 13:100159. doi: 10.1016/j.metop.2021.100159, PMID: 34938983 PMC8677426

[49] Lee S Lee J Cho S-H Roh G Park H-J Lee Y-J . Assessing the impact of mRNA vaccination in chronic inflammatory murine model. NPJ Vaccines. (2024) 9. doi: 10.1038/s41541-024-00825-z, PMID: 38360752 PMC10869740

[50] Schelldorfer A Gregoriano C Hauser S Fuchs TA Mueller B Schuetz P . Rate of cardiovascular events up to 8 years after uncomplicated myocarditis: a nationwide cohort study. Eur Heart J Acute Cardiovasc Care. (2024) 13:401–10. doi: 10.1093/ehjacc/zuae021, PMID: 38366232 PMC11132296

[51] Semenzato L Vu SL Botton J Bertrand M Jabagi M-J Drouin J . Long-Term prognosis of patients with myocarditis attributed to COVID-19 mRNA vaccination, SARS-COV-2 infection, or conventional etiologies. JAMA. (2024) 332:1367. doi: 10.1001/jama.2024.16380, PMID: 39186694 PMC11348078

[52] Murphy SP Kakkar R McCarthy CP Januzzi JL . Inflammation in heart failure. J Am Coll Cardiol. (2020) 75:1324–40. doi: 10.1016/j.jacc.2020.01.014, PMID: 32192660

[53] Su J-H Luo M-Y Liang N.- Gong S-X Chen W Huang W-Q . Interleukin-6: a novel target for Cardio-Cerebrovascular Diseases. Front Pharmacol. (2021) 12:745061. doi: 10.3389/fphar.2021.745061, PMID: 34504432 PMC8421530

[54] Petruk G Puthia M Petrlova J Samsudin F Strömdahl A-C Cerps S . SARS-CoV-2 spike protein binds to bacterial lipopolysaccharide and boosts proinflammatory activity. J Mol Cell Biol. (2020) 12:916–32. doi: 10.1093/jmcb/mjaa067, PMID: 33295606 PMC7799037

[55] Petrlova J Samsudin F Bond PJ Schmidtchen A . SARS-CoV-2 spike protein aggregation is triggered by bacterial lipopolysaccharide. FEBS Lett. (2022) 596:2566–75. doi: 10.1002/1873-3468.14490, PMID: 36050806 PMC9538650

[56] Wang H Yin J Gu X Shao W Jia Z Chen H . Immune regulator Retinoic Acid-Inducible Gene I (RIG-I) in the pathogenesis of cardiovascular disease. Front Immunol. (2022) 13:893204. doi: 10.3389/fimmu.2022.893204, PMID: 35693778 PMC9178270

[57] Tran DT Batchu SN Advani A . Interferons and interferon-related pathways in heart disease. Front Cardiovasc Med. (2024) 11:1357343. doi: 10.3389/fcvm.2024.1357343, PMID: 38665231 PMC11043610

[58] Chen H-J Tas SW De Winther MPJ . Type-I interferons in atherosclerosis. J Exp Med. (2019) 217. doi: 10.1084/jem.20190459, PMID: 31821440 PMC7037237

[59] Chen Y-R Chen C-L Kolz C Kang PT Lynch W DeLucia AL . Abstract 14877: Spike protein of SARS-COV-2 virus induces mitochondrial dysfunction in swine heart via redox impairment of HEME proteins and increasing superoxide generation. Circulation. (2023) 148. doi: 10.1161/circ.148.suppl_1.14877

[60] Greenberger JS Hou W Shields D Fisher R Epperly MW Sarkaria I . SARS-COV-2 spike protein induces oxidative stress and senescence in mouse and human lung. In Vivo. (2024) 38:1546–56. doi: 10.21873/invivo.13605, PMID: 38936937 PMC11215613

[61] Kulkovienė G Narauskaitė D Tunaitytė A Volkevičiūtė A Balion Z Kutakh O . Differential mitochondrial, oxidative stress and inflammatory responses to SARS-COV-2 spike protein receptor binding domain in human lung microvascular, coronary artery endothelial and bronchial epithelial cells. Int J Mol Sci. (2024) 25:3188. doi: 10.3390/ijms25063188, PMID: 38542162 PMC10969886

[62] Pileggi CA Parmar G Elkhatib H Stewart CM Alecu I Côté M . The SARS-CoV-2 spike glycoprotein interacts with MAO-B and impairs mitochondrial energetics. Curr Res Neurobiol. (2023) 5:100112. doi: 10.1016/j.crneur.2023.100112, PMID: 38020812 PMC10663135

[63] Münzel T Camici GG Maack C Bonetti NR Fuster V Kovacic JC . Impact of oxidative stress on the heart and vasculature. J Am Coll Cardiol. (2017) 70:212–29. doi: 10.1016/j.jacc.2017.05.035, PMID: 28683969 PMC5663297

[64] Peoples JN Saraf A Ghazal N Pham TT Kwong JQ . Mitochondrial dysfunction and oxidative stress in heart disease. Exp Mol Med. (2019) 51:1–13. doi: 10.1038/s12276-019-0355-7, PMID: 31857574 PMC6923355

[65] Wheeler SE Shurin GV Yost M Anderson A Pinto L Wells A . Differential antibody response to mRNA COVID-19 vaccines in healthy subjects. Microbiol Spectr. (2021) 9. doi: 10.1128/spectrum.00341-21, PMID: 34346750 PMC8552678

[66] Heo C-K Lim W-H Yang J Son S Kim SJ Kim D-J . Novel S2 subunit-specific antibody with broad neutralizing activity against SARS-CoV-2 variants of concern. Front Immunol. (2023) 14:1307693. doi: 10.3389/fimmu.2023.1307693, PMID: 38143750 PMC10749193

[67] Braun E Sauter D . Furin-mediated protein processing in infectious diseases and cancer. Clin Transl Immunol. (2019) 8. doi: 10.1002/cti2.1073, PMID: 31406574 PMC6682551

[68] Ramazanov BR Tran ML Von Blume J . Sending out molecules from the TGN. Curr Opin Cell Biol. (2021) 71:55–62. doi: 10.1016/j.ceb.2021.02.005, PMID: 33706234 PMC8328904

[69] Fan J Hu Z Zeng L Lu W Tang X Zhang J . Golgi apparatus and neurodegenerative diseases. Int J Dev Neurosci. (2008) 26:523–34. doi: 10.1016/j.ijdevneu.2008.05.006, PMID: 18599251

[70] Li H Sun S . Protein aggregation in the ER: calm behind the storm. Cells. (2021) 10:3337. doi: 10.3390/cells10123337, PMID: 34943844 PMC8699410

[71] Hollebeeck S Raas T Piront N Schneider Y-J Toussaint O Larondelle Y . Dimethyl sulfoxide (DMSO) attenuates the inflammatory response in the *in vitro* intestinal Caco-2 cell model. Toxicol Lett. (2011) 206:268–75. doi: 10.1016/j.toxlet.2011.08.010, PMID: 21878375

[72] Kloesch B Liszt M Broell J Steiner G . Dimethyl sulphoxide and dimethyl sulphone are potent inhibitors of IL-6 and IL-8 expression in the human chondrocyte cell line C-28/I2. Life Sci. (2011) 89:473–8. doi: 10.1016/j.lfs.2011.07.015, PMID: 21821055

[73] Han H Kang J-K Ahn KJ Hyun C-G . DMSO alleviates LPS-Induced inflammatory responses in RAW264.7 macrophages by inhibiting NF-κB and MAPK activation. BioChem. (2023) 3:91–101. doi: 10.3390/biochem3020007

[74] Yoon C Kim H Mishchenko N Vasileva E Fedoreyev S Stonik V . Spinochrome D attenuates Doxorubicin-Induced cardiomyocyte death via improving glutathione metabolism and attenuating oxidative stress. Mar Drugs. (2018) 17:2. doi: 10.3390/md17010002, PMID: 30577438 PMC6356724

[75] Wang Y Jasper H Toan S Muid D Chang X Zhou H . Mitophagy coordinates the mitochondrial unfolded protein response to attenuate inflammation-mediated myocardial injury. Redox Biol. (2021) 45:102049. doi: 10.1016/j.redox.2021.102049, PMID: 34174558 PMC8246635

[76] Marchese AM Rousculp M Macbeth J Beyhaghi H Seet BT Toback S . The Novavax Heterologous Coronavirus Disease 2019 booster demonstrates lower reactogenicity than messenger RNA: a Targeted review. J Infect Dis. (2024) 230:e496–502. doi: 10.1093/infdis/jiad519, PMID: 37992183 PMC11326839

