# Supplementary material for: mRNA-based SARS-CoV-2 vaccines: intracellular processing and aggregation of the encoded spike protein as a mechanistic contributor to cardiac cellular stress
Source: Front Immunol. 2026 Feb 20;17:1635478. doi: 10.3389/fimmu.2026.1635478 (PMC12963247; doi:10.3389/fimmu.2026.1635478)
Supplement: Supplementary file 1 [file DataSheet1.pdf]

Original Research Article

mRNA-based SARS-CoV-2 vaccines: intracellular processing and aggregation of the encoded spike protein as a mechanistic contributor to cardiac cellular stress

Rolf Schreckenberg, Nadine Woitasky, Nadja Itani, Laureen Czech, Anita C. Windhorst, Malte Juchem, Christian Bär, Thomas Thum, Péter Ferdinandy, Rainer Schulz

Supplementary material

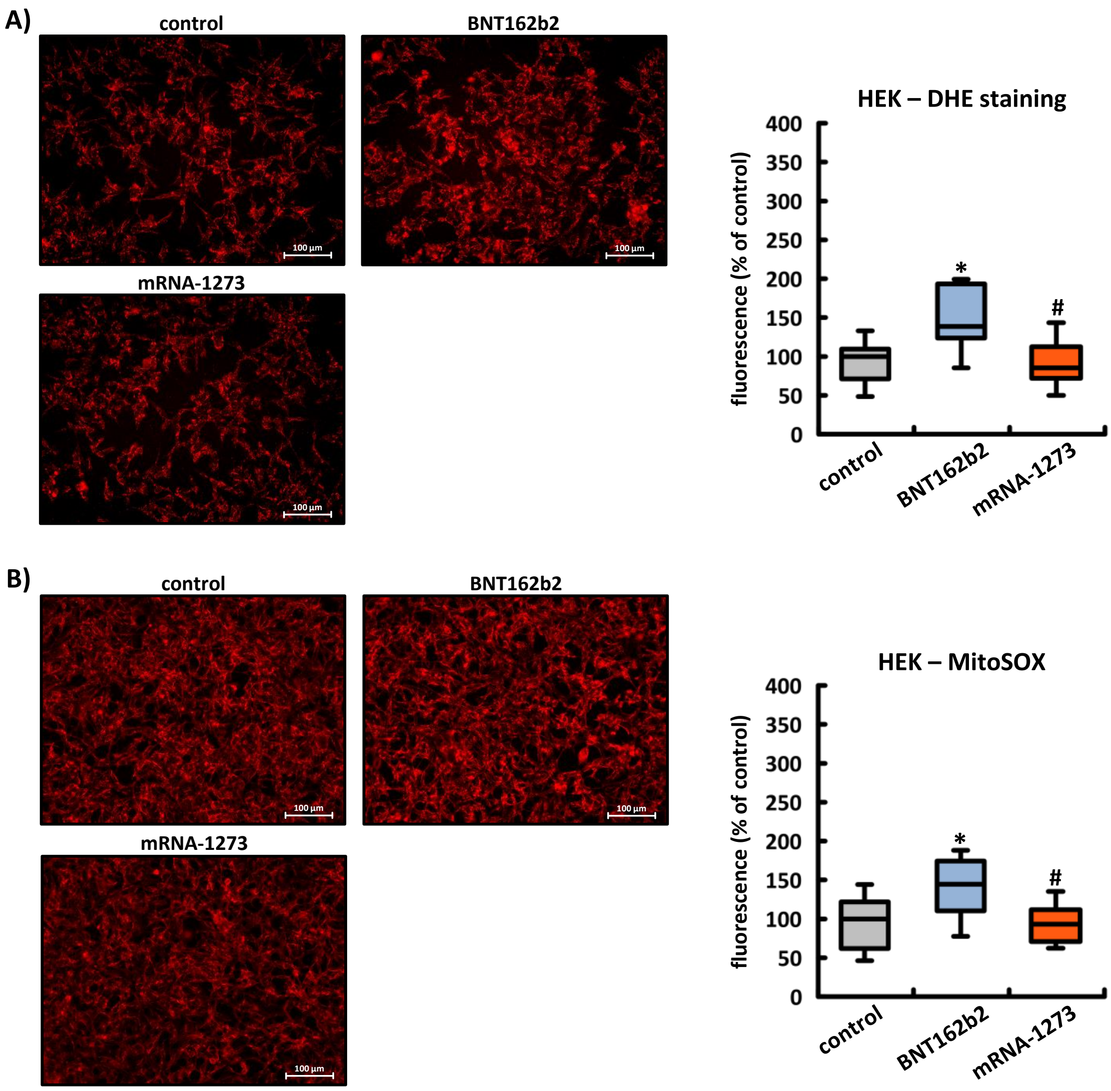

Fig. S1: ***The influence of BNT162b2 and mRNA-1273 on superoxide production in HEK-293 cells.*** Fluorescence microscopic images of HEK-293 cells whose degree of oxidative stress was measured using the two superoxide indicators DHE **(A)** and MitoSOX **(B)**. Reactive oxygen species were always detected at 24 h in untreated control cells and after application of BNT162b2 or mRNA-1273. The results are based in each case on n=5 culture dishes from n=5 cell passages; three image sections per culture dish were recorded densitometrically. *, p< 0.05 vs. control, #, p< 0.05 vs. BNT162b2.

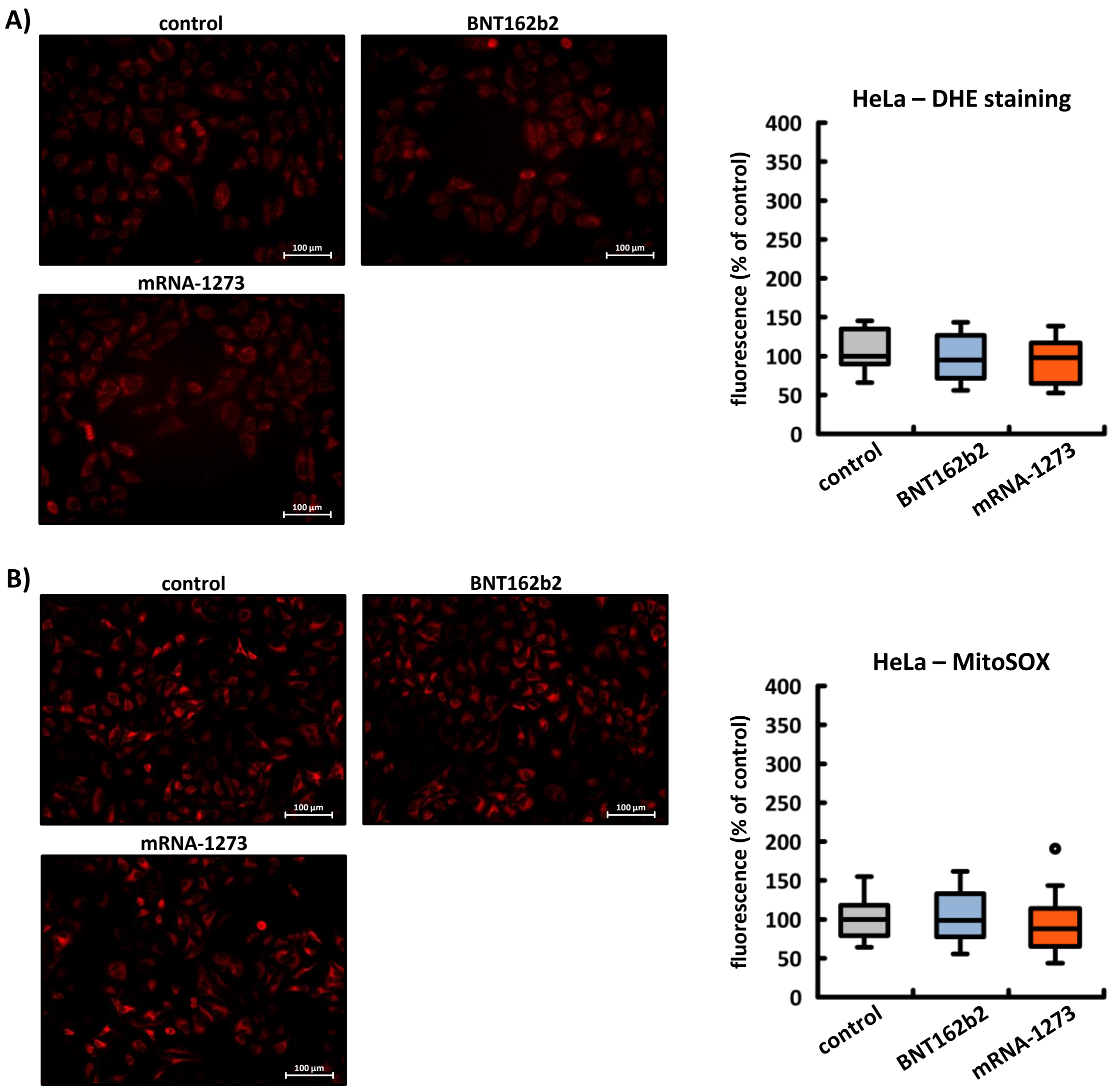

Fig. S2: ***The influence of BNT162b2 and mRNA-1273 on superoxide production in HeLa cells.*** Fluorescence microscopic images of HeLa cells whose degree of oxidative stress was measured using the two superoxide indicators DHE **(A)** and MitoSOX **(B)**. Reactive oxygen species were always detected at 24 h in untreated control cells and after application of BNT162b2 or mRNA-1273. The results are based in each case on n=5 culture dishes from n=5 cell passages; three image sections per culture dish were recorded densitometrically.

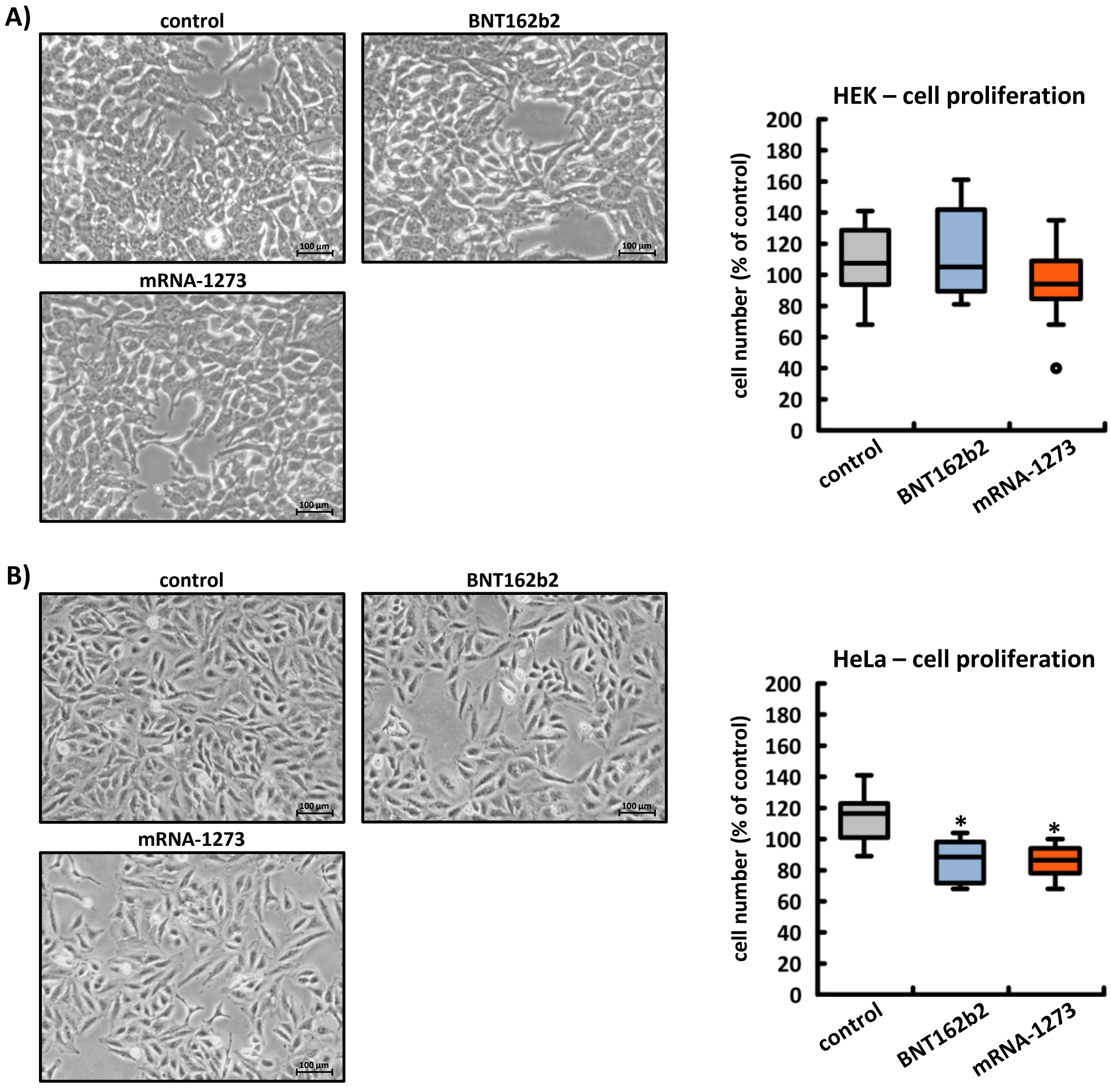

Fig. S3: The effects of BNT162b2 and mRNA-1273 on cell proliferation. An Olympus CKX41 was used to take photographs at 24 h of untreated control cells and cells following application of BNT162b2 or mRNA-1273 on **A)** HEK-293 and **B)** HeLa cells. The cells in two identically sized image sections per culture dish were counted using BZ Advanced Analysis Software (Keyence Corporation, version 3.60). The results are based in each case on n=5 culture dishes from n=5 cell passages. *, $p < 0.05$ vs. control.

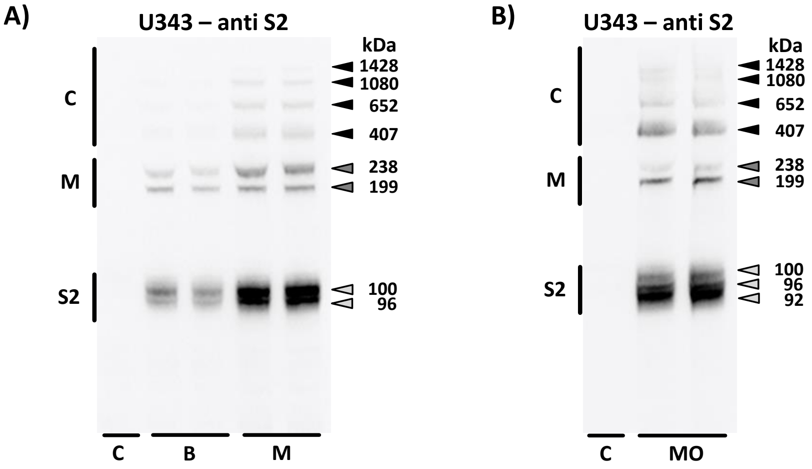

Fig. S4: ***Translation of the encoded spike monomers in U343 cells.*** The figure shows all spike protein associated products in U343 astrocytes detected through the S2 antibody 24 h after application of **A)** BNT162b2 (B) and mRNA-1273 (M) as well as **B)** mRNA-1273 222 (MO). The band pattern could be reproduced on cells from n=2 cell passages. C = untreated controls.
(S2 = S2 subunit, M = spike monomers, C = complexes of spike monomers and its subunits)

The astrocytoma cell line U343 was kindly provided by Prof. Wartenberg (Department of Internal Medicine I, Friedrich Schiller University Jena). Cells were cultured using the same procedure as for HEK-293 and HeLa cells (see Methods).

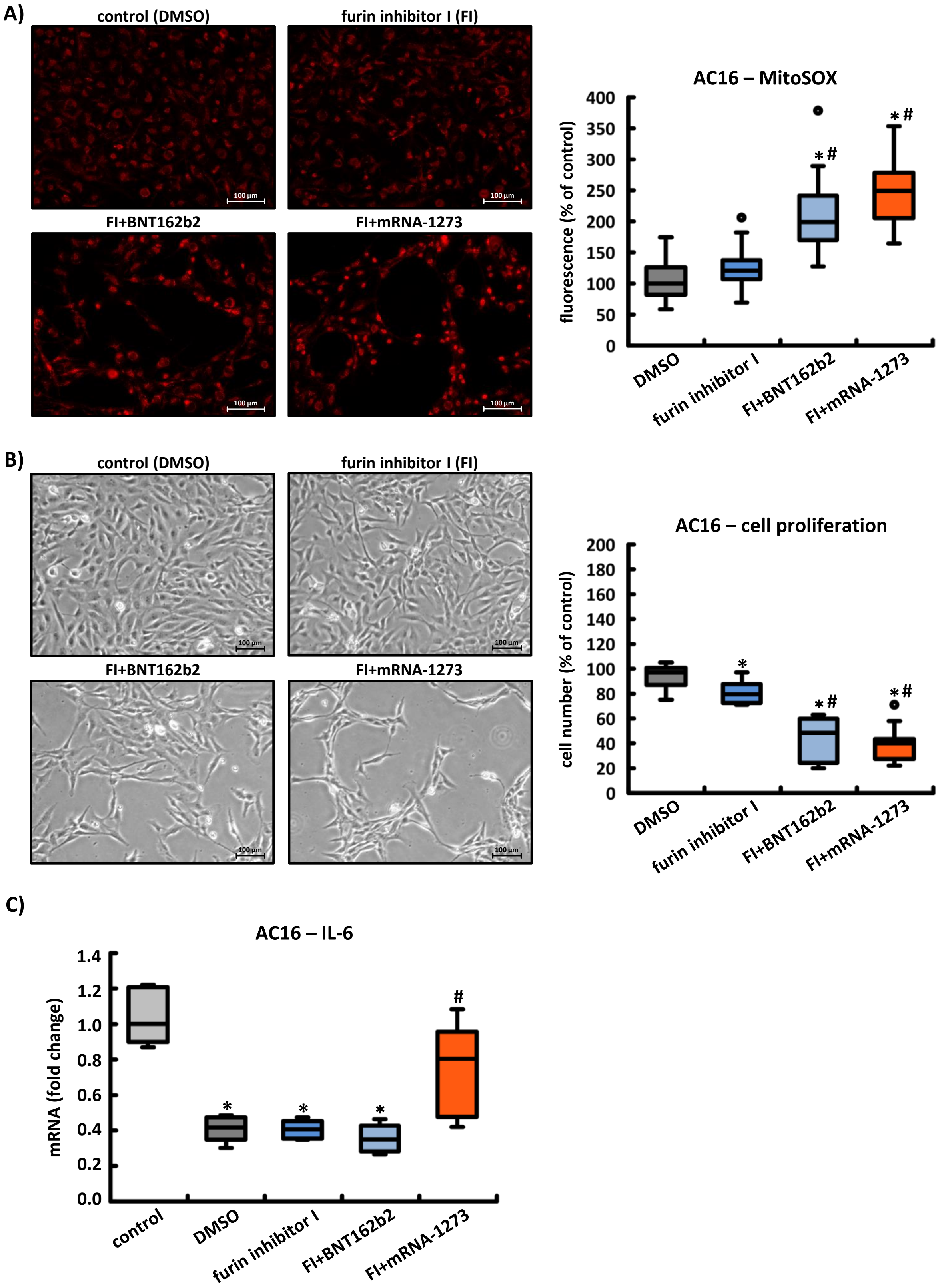

Fig. S5: Effects of furin-dependent spike processing on cellular stress parameters. A) Fluorescence microscopic images of cardiac AC16 cells whose degree of oxidative stress was measured at 24 h using the superoxide indicator MitoSOX. The furin inhibitor I (FI), applied in dimethyl sulphoxide (DMSO, final concentration 4 µl/ml cell culture medium), induced superoxide production by 21% (not significant), whereas BNT162b2 and mRNA-1273 in FI-preincubated cells led to a 1.98x (± 0.60) and 2.49x (± 0.53) increase in relative fluorescence, respectively. The analysis is based on n=6 culture dishes from n=3 cell passages; three image sections per culture dish were recorded densitometrically. **B)** Under furin

inhibition, a significant reduction in cell proliferation was already detectable after 24 h; this effect was further enhanced by subsequent application of either BNT162b2 or mRNA-1273. Two identically sized image sections per culture dish were evaluated. The analysis is based on n=6 culture dishes from n=3 cell passages. *, $p < 0.05$ vs. DMSO, #, $p < 0.05$ vs. furin inhibitor I. Since no differences were found between the untreated and DMSO controls in the MitoSOX and proliferation assays, only the DMSO control is shown for these conditions. **C)** At 24 h, IL-6 expression was reduced to 42% (0.42 ± 0.07) compared to untreated control cells following DMSO application. Under these anti-inflammatory conditions, a significant increase in IL-6 expression to 80% (0.80 ± 0.23) was observed only after treatment with mRNA-1273. The analysis is based on n=6 culture dishes from n=3 cell passages. *, $p < 0.05$ vs. control, #, $p < 0.05$ vs. DMSO.

Targets	Accession Number	Forward Sequence	Reverse Sequence
HPRT	NM_000194.3	CCTGGCGTCGTGATTAGTGA	CGAGCAAGACGTTCAGTCCT
IL-6	NM_000600.5	AGTGAGGAACAAGCCAGAGC	AGCTGCGCAGAATGAGATGA
IFIT1	NM_001548.5	CTCTGCCTATCGCCTGGATG	AGCTTCAGGGCAAGGAGAAC
RIG-I	NM_014314.4	TGTCCACCTTCAGAAGTGTCT	CCCCTTTTGTCCTTGTGGGA
BNT162b2	Jeong et al. (1)	GGATCCTCTGAGCGAGACAA	ACAGGTCGTTCAGCTTGGTA
mRNA-1273	Jeong et al. (1)	GCCTACAGCAACAACAGCAT	TTGAACAGCAGGTCCTCGAT

Table S1: Information on PCR Primers

Above we provide the names of the primer pairs along with their corresponding accession numbers and sequences. The primers for BNT162b2 and mRNA-1273 were designed based on publicly available assemblies of the SARS-CoV-2 spike-encoding mRNA sequences (1).

Reference

1. Jeong DE, McCoy M, Artiles K, Ilbay O, Fire A, Nadeau K, et al. Assemblies of putative SARS-CoV-2-spike-encoding mRNA sequences for vaccines BNT-162b2 and mRNA-1273. Available online at: https://virological.org/t/assemblies-of-putative-sarscov2-spike-encoding-mrna-sequences-for-vaccines-bnt-162b2-and-mrna-1273/663 (accessed December 2024).
The sequence documents referenced for primer design have not been updated since 2021 and remained unchanged until at least December 2024.
